# Achieving Wide-Temperature-Range Physical and Chemical Hydrogen Sorption in a Structural Optimized Mg/N-Doped Porous Carbon Nanocomposite

**DOI:** 10.1007/s40820-025-01931-w

**Published:** 2026-01-02

**Authors:** Yinghui Li, Li Ren, Zi Li, Yingying Yao, Xi Lin, Wenjiang Ding, Andrea C. Ferrari, Jianxin Zou

**Affiliations:** 1 Shanghai Key Laboratory of Hydrogen Science & Center of Hydrogen Science, Shanghai Jiao Tong University, Shanghai, 200240 People’s Republic of China; 2 National Engineering Research Center of Light Alloys Net Forming & State Key Laboratory of Metal Matrix Composites, Shanghai Jiao Tong University, Shanghai, 200240 People’s Republic of China; 3 Cambridge Graphene Centre, University of Cambridge, Cambridge, CB3 0FA UK

**Keywords:** Hydrogen storage, MgH_2_, Porous carbon, Nanoconfinement, Physi- and chemisorption

## Abstract

Nanoconfinement is a promising approach to simultaneously enhance the thermodynamics, kinetics, and cycling stability of hydrogen storage materials. The introduction of supporting scaffolds usually causes a reduction in the total hydrogen storage capacity due to “dead weight.” Here, we synthesize an optimized N-doped porous carbon (rN-pC) without heavy metal as supporting scaffold to confine Mg/MgH_2_ nanoparticles (Mg/MgH_2_@rN-pC). rN-pC with 60 wt% loading capacity of Mg (denoted as 60 Mg@rN-pC) can adsorb and desorb 0.62 wt% H_2_ on the rN-pC scaffold. The nanoconfined MgH_2_ can be chemically dehydrided at 175 °C, providing ~ 3.59 wt% H_2_ with fast kinetics (fully dehydrogenated at 300 °C within 15 min). This study presents the first realization of nanoconfined Mg-based system with adsorption-active scaffolds. Besides, the nanoconfined MgH_2_ formation enthalpy is reduced to ~ 68 kJ mol^−1^ H_2_ from ~ 75 kJ mol^−1^ H_2_ for pure MgH_2_. The composite can be also compressed to nanostructured pellets, with volumetric H_2_ density reaching 33.4 g L^−1^ after 500 MPa compression pressure, which surpasses the 24 g L^−1^ volumetric capacity of 350 bar compressed H_2_. Our approach can be implemented to the design of hybrid H_2_ storage materials with enhanced capacity and desorption rate.

**Supplementary Information:**

The online version contains supplementary material available at 10.1007/s40820-025-01931-w.

## Highlights

- The as-synthesized rN-pC exhibited H_2_ uptake of ~0.9 wt% at 77 K and ultralow pressure of ~0.1 bar, with an isosteric adsorption enthalpy (Q_st_) of ~14 kJ mol^-1^ H_2_ at zero coverage.
- The 60MgH_2_@rN-pC started to decompose at 175 °C and released H_2_ of 3.38 wt% at 300 °C within 30 min, which showed outstanding desorption kinetics of MgH_2_ among Mg-carbon material nanocomposites.
- The drawback of nanoconfinement scaffolds that cannot store hydrogen was firstly overcome.

**Supplementary Information:**

The online version contains supplementary material available at 10.1007/s40820-025-01931-w.

## Graphical abstract

**Figure:**
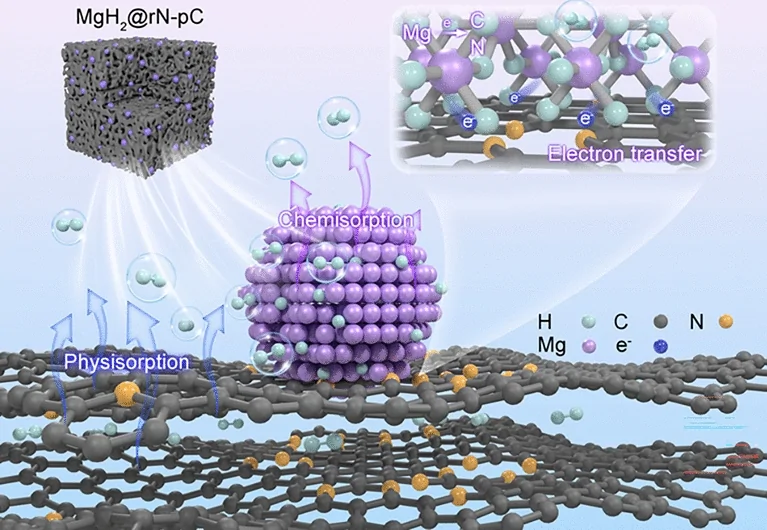

## Introduction

Clean and renewable energy is vital for sustainable development [1]. Hydrogen has high gravimetric energy density (lower heating value (LHV) ~ 120 MJ kg^−1^) [2] and zero carbon emission (only water as combustion product) [3], making it promising as energy carrier. However, the large-scale application of H_2_ as energy carrier is still hampered by the difficulties of effective storage [4]. Compared with storing H_2_ as a compressed gas at high pressures up to 70 MPa [5], or as a liquid at cryogenic temperatures (T = 20 K) [6], solid-state H_2_ storage has the advantages of mild operation conditions (lower than 10 Mpa working pressure [7] and higher than 77 K working T [8] for storage and transportation) and reduced cost of storage systems. Due to the high gravimetric and volumetric H_2_ storage density (~ 7.6 wt% H_2_ and ~ 110 kg m^−3^ H_2_, respectively) [9], complete heat-driven reversible transformation (MgH_2_ ⇌ Mg + H_2_) [10], and earth-abundant natural Mg containing minerals such as dolomite [11] and seawater [12], MgH_2_ is one of the most promising candidates for solid-state H_2_ storage [13]. Nonetheless, the high thermodynamic stability and kinetic reaction barriers (ΔH =  ~ 75 kJ mol^−1^ H_2_, and ΔE =  ~ 160 kJ mol^−1^ H_2_, respectively) [14] limit its industrial application. Zhang et al. studied the solar-driven reversible hydrogen storage of MgH_2_ and utilized solar energy as the sustainable energy source to reduce the energy costs [15, 16]. Besides, different approaches have been proposed to improve the thermodynamic and kinetic properties of MgH_2_, including alloying [17], catalysts doping [18], and nanostructuring [19].

Nanostructuring (reducing particle size to the nanoscale or confining of nanoparticles (NPs) in supporting materials) of MgH_2_ is a promising approach to enhance its thermodynamic and kinetic performances simultaneously [20], since this results in heterostructured interfaces with enhanced energy, shortened H diffusion pathways, and increased active sites [21]. Nanoconfinement of Mg/MgH_2_ NPs in porous supporting scaffolds is an effective way to synthesize nanosized Mg-based H_2_ storage materials [22]. The porous supporting materials could prevent Mg/MgH_2_ NPs aggregation and growth during thermal-induced de/re-hydrogenation cycles, usual for free-standing nanostructured Mg/MgH_2_ [23]. An excellent supporting scaffold should have chemical inert (stability under operation T and H_2_ atmosphere), light weight (low volumetric density) and catalytic activity for the H_2_ ab/desorption process. Carbon-based materials, such as graphene [24], carbon aerogels [25], ordered mesoporous carbon (CMK3) [26] et al. have been used as supporting materials to confine MgH_2_ NPs. Xia et al. prepared monodisperse MgH_2_ NPs self-assembled on Ni-modified graphene with multilayer synthesized through chemical intercalation of graphite. Hydrogenated composites could realize complete dehydrogenation at 200 °C within 150 min [24]. Jensen et al. synthesized nanoporous carbon aerogel scaffolds X1 and X2, with average pore sizes of 22 and 7 nm, and the dehydrogenation kinetics of the confined hydride in smaller pores was faster due to the size reduction [25]. However, these carbon-based materials show no catalytic activity [27], and additional metal catalysts, such as Fe, Co, Ni [28, 29], are needed to improve the H_2_ storage performance. The loading of MgH_2_ is limited by the scaffolds’ structure [25], and the synthesis routes [30]. Pore blocking was found in oxygen-containing carbons, and bulk Mg was detected in the samples with Mg loadings > 15 wt% [31], indicating loading should be selected considering the compromise between capacity and desorption T [32]. Besides, the introduction of a scaffold without H_2_ storage capacity leads to the loss of total H_2_ storage capacity. It is thus necessary to prepare a scaffold with self-adsorbing H_2_ capacity for nanoconfinement of Mg/MgH_2_ NPs, so as to improve the Mg-based H_2_ storage performance and introduce extra physical H_2_ adsorption capacity.

The development of high-pressure and cryogenic technologies (with controllable operation over 10 MPa at −196 °C) [33] enables the exploration of new adsorbent materials, such as metal organic frameworks (MOFs) [34], covalent organic frameworks (COFs) [35], and porous carbons (pCs) [36], as other promising strategies to realize safe and cost-effective H_2_ storage. Among them, porous carbons have the advantages of easy synthesis, thermal and chemical stability, and tuneable porosity, as well as being suitable confinement scaffolds for Mg/MgH_2_ [37].

Here, we synthesize an ammonia (NH_3_)-optimized N-doped porous carbon (rN-pC) with reversible H_2_ adsorption capacity to confine MgH_2_. For H_2_ adsorption a high zero coverage adsorption heat (= 14 kJ mol^−1^ H_2_) was achieved, leading to adsorption capacity close to 1% at a pressure as low as 0.01 Mpa. Mg NPs form on rN-pC to obtain a 60 Mg@rN-pC composite through nanoconfinement. N-doped carbon facilitates electron transfer from MgH_2_ to the scaffold, weakening the Mg–H bonds and decreasing the operation T for dehydrogenation. 60MgH_2_@rN-pC shows low onset desorption temperature (T_onset_, 175 °C) and 10-cycling H_2_ absorption and desorption stability, presenting outstanding catalytic effects when compared to state-of-art non-metal catalyzed MgH_2_ systems [38]. 60MgH_2_@rN-pC composites can deliver 4.2 wt% H_2_ within two T ranges (physical desorption from -196 °C and chemical desorption from 175 °C). As T increases, the H_2_ stored in the micropores of scaffold (N–pC) by physical adsorption at −196 °C is preferentially released, followed by further release of H_2_ stored in MgH_2_ via chemisorption. Thus, our synergistic approach yields a hybrid H_2_ storage composite material, with both physical and chemical H_2_ sorption ability, enabling a new strategy to develop advanced energy storage materials.

## Experimental Section

### Materials

Zinc nitrate hexahydrate (Zn(NO_3_)0.6H_2_O 98%) was purchased from Thermo Fisher Scientific Inc. (USA). 2-methylimidazole (2-Hmim98%) and methanol were supplied by Shanghai Aladdin Biochemical Technology Co., Ltd. (China). Anhydrous tetrahydrofuran (THF, 99.5%) was purchased from Tokyo Chemical Industry (TCI, Japan). Methyl magnesium chloride (CH_3_MgCl, 3 M solution in anhydrous THF) and lithium foil (Li, 99.9%) were purchased from Sigma-Aldrich Lab (USA). Naphthalene (C_10_H_8_, > 99.7%,) was supplied by Shanghai Macklin Biochemical Co., Ltd. (China).

### Synthesis of rN-pC Scaffolds

#### Synthesis of rN-pC Scaffolds

2.97 g Zn(NO_3_)0.6H_2_O and 6.57 g 2-Hmim are dissolved in 200 mL of methanol, respectively. These two solutions are mixed and agitatedly stirred (500 rpm) for 10 min, followed by standing for 24 h at room temperature (RT). The resultant white precipitation is centrifuged and washed 3 times with methanol, and dried in an oven at 60 °C overnight to obtain ZIF-8 NPs. ZIF-8 is heated to 1000 °C with a ramp of 10 °C min^−1^ under Ar/5% H_2_ and kept at 1000 °C for 30 min (denoted as N-pC), followed by NH_3_ treatment at 750 °C for 30 min. rN-pC is obtained after cooling to RT.

#### ***Synthesis of 60MgH***_***2***_***@rN-pC***

All samples are prepared in an inert atmosphere (Ar-filled glove box, Mikrouna). 60MgH_2_@rN-pC is synthesized by a reduction reaction of in situ grown Mg NPs on rN-pC. CH_3_MgCl, Li foil, C_10_H_8_ and anhydrous THF are used as received without further treatment. 30 mg as-prepared rN-pC was processed in 20 mL THF under intermittent ultrasonication for 1 h, and then 0.6 mL CH_3_MgCl was added for another 1 h intermittent sonication (200 W for 2 h, 15 s pulse and 15 s relaxation) (solution A). 31.23 mg Li foil and 615.26 mg naphthalene were dissolved in 30 mL THF with rapid stirring (800 rpm) for 3 h (solution B). Then, solution A was dripped dropwise into solution B and stirred for 2 h at RT. 0.6 mL of CH_3_MgCl (1.8 mmol) can be reduced to get 43.2 mg Mg and hydrogenated to obtain 46.8 mg MgH_2_. Therefore, the weight percent of MgH_2_ in the 60MgH_2_@rN-pC composite is calculated to be ~ 60 wt% (46.8/76.8 mg). The 60 Mg@rN-pC composite is derived from the desorption of 60MgH_2_@rN-pC. The resultants were centrifugated and washed 3 times with THF. Finally, the participants were dried under vacuum in the glove box and hydrogenated to obtain 60MgH_2_/rN-pC. Pure Mg/MgH_2_ were synthesized with the same steps without adding rN-pC. In the present research, 60MgH_2_@rN-pC-as synthesis/absorption/desorption belong to different states of one sample.

### Materials Characterization

Specific surface area (SSA) and pore size distribution (PSD) are estimated via N_2_ ad/desorption tests at −196 °C on a Autosorb-IQ3 apparatus (Quantachrome). The phase composition is analyzed by X-ray diffraction (XRD, Mini Flex 600, Rigaku, Cu Kα) at 40 kV/15 mA with a scanning speed of 10° min^−1^. The samples are prepared in an Ar-filled glovebox and sealed in a custom-designed holder covered by an amorphous tape to avoid air exposure during testing. (High-resolution) Transmission electron microscopy ((HR)TEM) and selected area electron diffraction (SAED) are performed on a Talos F200X G2 microscope with an accelerating voltage of 200 kV. The samples were ultrasonically dispersed in tetrahydrofuran (THF), and then, in a glovebox, the dispersion liquid was dropped on a copper grid, which was sealed and transferred to the TEM instrument rapidly. The morphologies of products are also examined by scanning electron microscopy (SEM, Mira3 and Rise-magna, TESCAN). An air-proof transfer vessel is used to transfer the samples from glovebox to X-ray photoelectron spectroscopy equipment (XPS, Kratos AXIS Ultra DLD, Al Kα/Mg Kα) to characterize the elemental valence and chemical bonding. For the XPS analysis of powder samples, the following preparation procedures were employed: In an argon-filled glovebox, a minimal amount of sample was transferred onto conductive carbon tape mounted on the sample holder using a toothpick, followed by flattening the surface with a spatula. The whole sample stage was sealed in the air-proof transfer vessel, which would be connected with the testing instrument directly. For the XPS measurements, monochromatic X-ray of Al Kα (~ 1487 eV) was used to induce the generation of photoelectrons. For the comparison of one element in different samples, the same testing parameters (working steps, dwell times, etc.) were used to minimize systematic errors. The C–C binding energy at 284.8 eV is used to calibrate the XPS data [39]. Raman spectra are collected using micro-Raman spectroscopy from Renishaw inVia Qontor with 532 nm lines and from Horiba LabRAM HR Evolution, Horiba Scientific with 325, 514, and 633 nm. FTIR spectra in transmittance mode are measured with a Thermo Scientific Nicolet iS5. DSC curves are obtained on a NETZSCH STA449F3 Jupiter facility under flowing Ar. The measurements were conducted by heating the samples from room temperature to 500 °C with the heating rates of 3, 5, 7, and 10 °C min^−1^. Thus, the testing duration varies with the heating rate, which is 158.3, 95, 67.8, and 47.5 min, for the heating rates of 3, 5, 7, and 10 °C min^−1^, respectively. Around 5–10 mg sample was used for each test. Activation energy (E_a_) is obtained by fitting DSC data using the Kissinger method. The isothermal ad/ab/desorption tests and TPD measurements are carried out in a Sievert-type pressure-composition-T apparatus (PCT, HPSA-auto device, China), in which the modified Benedict–Webb–Rubin (MBWR) [40] equation of state (EOS) is used to calculate H_2_ storage capacity. −196/−186 °C isothermal volumetric H_2_ adsorption tests are done at liquid N_2_ and Ar, respectively. Before the physical adsorption measurements, all samples are outgassed at 300 °C for 12 h, and then the Mg is fully adsorbed at 300 °C. Subsequently, the samples are cooled down to room temperature under certain H_2_ back pressure and then degassed in vacuum for another 1 h. Typically, for the isothermal ab/desorption tests, ~ 100 mg is loaded into the test tube in a glove box and heated to the preset T by rapid heating rate (~ 5–10 °C min^−1^), then maintaining this T during the thermodynamic test. The H_2_ ab/desorption kinetics is measured at various T for an initial H_2_ pressure of 3 MPa for absorption and 0.001 MPa for desorption, respectively. The hydrogenated samples are heated from RT to 350 °C with a heating rate ~ 2 °C min^−1^ under a primary vacuum to conduct TPD measurements. The H_2_ pressure/duration/T parameters of pure Mg used for cycling tests are set as 3 MPa/20 min/350 °C for re-hydrogenation and 0.03 MPa/45 min/350 °C for dehydrogenation. For 60MgH_2_/rN-pC those are set to 3 MPa/15 min/275 °C for re-hydrogenation and 0.03 MPa/60 min/275 °C for dehydrogenation. H_2_ storage capacity is calculated as wt% of entire composite including rN-pC scaffold. The compression machine is a PC-12 type tablet press from Tianjin Jingtuo Company. A cylindrical die with the inner diameter 10 mm was used to prepare the composite pellets and the effective pressure on the pellets was set at ~ 500 MPa (4 Tons).

### Density Functional Theory Calculations

Density functional theory (DFT) calculations are performed using quantum espresso (QE) based on the pseudopotential plane wave (PPW) method [41]. Perdew–Burke–Ernzerhof (PBE) functionals [42] are used to describe exchange–correlation effects of electrons. The projected augmented wave (PAW) potentials [43] are used to describe the ionic cores and take valence electrons into account using a plane wave basis set with a kinetic energy cutoff = 500 eV. All structures are first optimized to reach their most stable configuration. During geometry optimization, all atom positions are allowed to relax. Brillouin-zone sampling is conducted using a Monkhorst–Pack (MP) [44] grid with separation of 0.04 Å^−1^. The convergence criterion for the electronic self-consistent field (SCF) loop is set to 1 × 10^–5^ eV atom^−1^ to ensure accurate total energy calculations while maintaining computational efficiency [45]. The atomic structures are optimized until the residual forces are < 0.05 eV Å^−1^. The adsorption energies between porous carbon substrates and H_2_ are computed as [46]:

$${E}_{ad}={E}_{sub-H2}-{E}_{sub}-{E}_{H2},$$

The differential charge density is calculated as [46]: $\Delta \rho ={\rho }_{tot}-{\rho }_{sub}-{\rho }_{abs}$.

where ${\rho }_{tot}$, ${\rho }_{sub}$ and ${\rho }_{abs}$ represent the total charge density, charge density of substrate and charge density of absorbed molecules.

## Results and Discussion

### Preparation and Characterization of rN-pC

MOFs have high SSA from hundreds to thousands m^2^ g^−1^ [47, 48], but relatively poor thermal stability compared with zeolites [49] and carbons [50], so their degassing pre-activation is difficult and sensitive to T control. The thermal stability of carbon materials derived from MOFs has improved with the decomposition and carbonization of ligands [51], but their SSA has reduced due to the collapse of the ordered three-dimensional structures during heat treatment [52]. NH_3_ etching of the carbon matrix leads the formation of pores, as well as the introduction of active N atoms, favorable for H_2_ adsorption [53]. Here, we prepared N-doped porous carbon (N-pC) from zeolitic imidazolate frameworks (ZIF-8) by heat treatment. Its structure and composition are optimized by NH_3_ treatment to enrich N doping porous carbon (rN-pC) (Fig. S1).

**Fig. 1:**
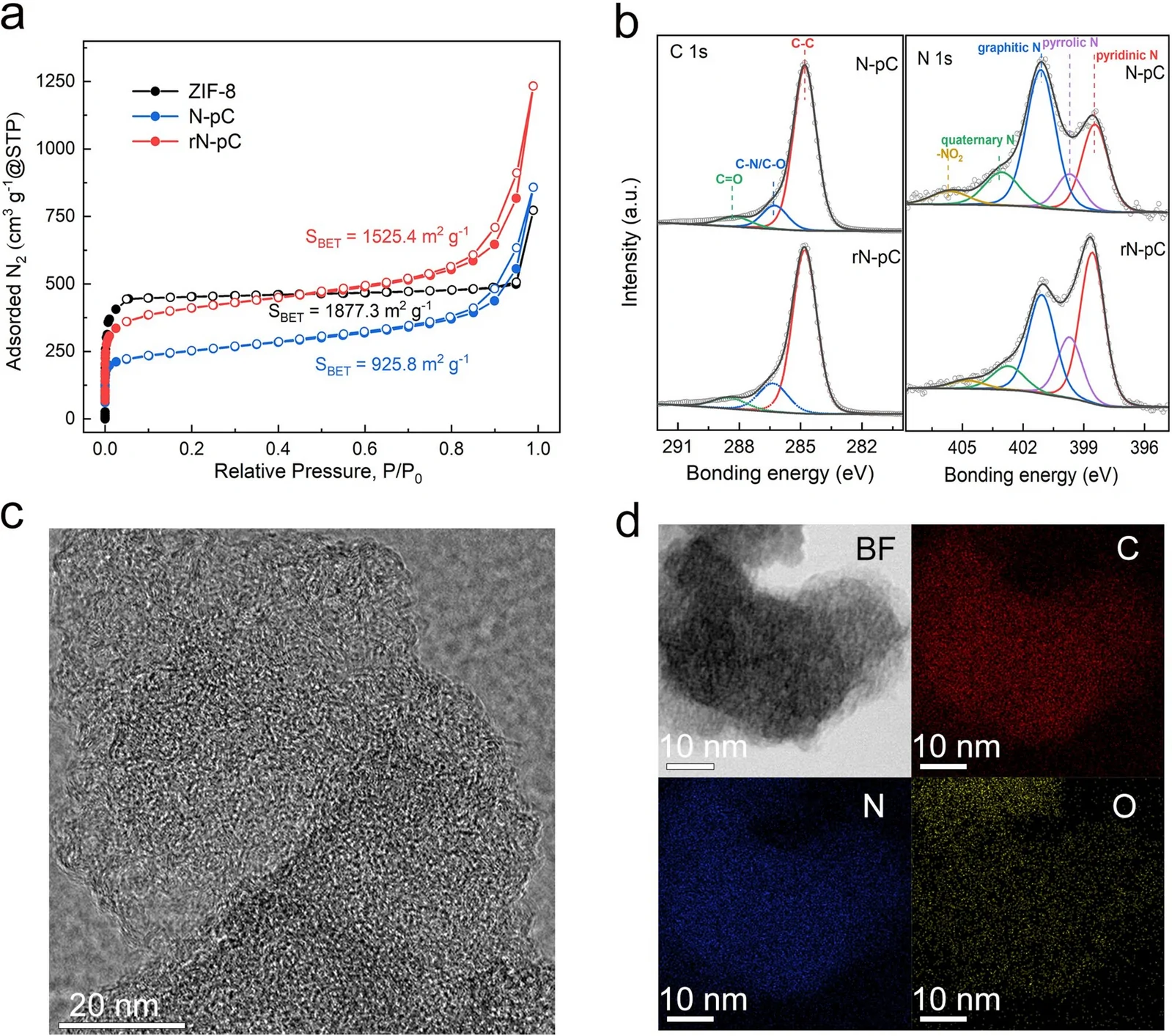
Structure and morphology characterization of rN-pC. **a** N_2_ adsorption/desorption isotherms, and **b** high-resolution XPS spectra of C 1*s* and N 1*s* of N-pC and rN-pC. **c** TEM image, **d** representative BF image and EDS elemental mappings of rN-pC

The crystal structures, pore distributions and heteroatoms are systematically characterized. X-ray diffraction (XRD) patterns in Fig. S2a of the as-synthesized ZIF-8 match the simulated XRD standard pattern and those reported in literatures [54, 55]. After pyrolysis of ZIF-8 at 1000 °C, the resulting N-pC displays two humps at ~ 23.8° and 43.3°, implying its deficiency of periodic graphitic structure (full width at half maximum of (FWHM) (002) diffraction peak of ~ 12°) [56], while NH_3_ has little effect on the crystal structure (Fig. S2a). However, as shown in Figs. 1a, S2b and Table S1, the SSA, PSD, and pore volume (PV) of N-pC change after NH_3_ treatment. Brunauer–Emmett–Teller (BET) tests in Fig. 1a show SSA and PV of ZIF-8 ~ 1877 m^2^ g^−1^ and 0.71 cm^3^ g^−1^, much higher than N-pC (925.8 m^2^ g^−1^ and 0.92 cm^3^ g^−1^) owing to the structural collapse during pyrolysis. Correspondingly, N-pC has a type-IV isotherm [57], with an adsorption–desorption hysteresis loop at the relative pressure p/p_0_ of 0.5–1, suggesting the generation of mesopores (2–50 nm) [58], suitable for the impregnation of organic Mg precursors. SSA and PV of rN-pC increase to 1525.4 m^2^ g^−1^ and 1.50 cm^3^ g^−1^, mainly due to NH_3_ etching, thus achieving pore enlargement [59]. PSD plots are obtained using the Barrett–Joyner–Halenda (BJH) method to analyze the pore structure of N-pC and rN-pC (Fig. S2b). N-pC and rN-pC possess hierarchically porous architecture with additional mesopores compared with monosized microporous ZIF-8, while the pore structure changes from micro to meso.

Chemical compositions and elemental valence states are investigated by X-ray photoelectron spectroscopy (XPS) (Figs. 1b and S2c). XPS spectra of N-pC and rN-pC show three dominant peaks at 288, 399, and 532 eV, assigned to C 1*s*, N 1*s*, and O 1*s* [60]. The C 1*s* spectra are almost the same for both N-pC and rN-pC, and the high-resolution C 1s peak can be deconvoluted into C–C (284.8 eV), C-N/C-O (287.1 eV), and C = O (289.5 eV) groups [61]. The high-resolution N 1*s* XPS consists of 5 peaks with binding energies of 398.5, 399.7, 401.1, 402.9, and 405.0 eV, corresponding to pyridinic, pyrrolic, graphitic, quaternary, and oxidized N groups [59]. Compared with N-pC, rN-pC contains more pyridinic N groups, improving its electron-transferring ability [62]. From XPS (Table S2), the N content of rN-pC increases from 2.5 wt% to 6 wt%, suggesting the possible transformation of C = C to C-N during NH_3_ treatment. The Raman spectra were measured under different excitation of 325, 514, 532, and 633 nm with a linear baseline subtraction and then deconvoluted to Gaussian lines. Considering of the amorphous structure and broadening D peaks, the ratio of peak areas was compared [63]. The signals of N-pC and rN-pC were detected at ~ 1360 cm^−1^ (D band) and ~ 1580 cm^−1^ (G band), usually involving in the in-plane bond-stretching motion of all pairs of *sp*^*2*^ atoms and breathing mode of *sp*^*2*^ atoms in rings [64], respectively. 514 nm Raman spectra in Fig. S2d show that N-pC has D peak at ~ 1359 and G peak at ~ 1584 cm^−1^, with FWHM (G) = 91 cm^−1^ and I(D)/I(G) = 2.11, and rN-pC has D peak at ~ 1355 and G peak at ~ 1585 cm^−1^, with FWHM (G) = 113 cm^−1^ and I(D)/I(G) = 2.66. After NH_3_ treatment, the dilated G peak width and increasing values of I(D)/I(G) can be attributed to the N doping and activation of sixfold aromatic rings, as well as NH_3_ etching and shrinking of ring clusters, which is coincident with the BET and XPS results [65]. The results under different excitation wavelength exhibit the same trend in FWHM (G) and I(D)/I(G), which are shown in Fig. S3a-d with error bars.

The microstructure and morphology of ZIF-8, N-pC and rN-pC are further characterized by scanning electron microscopy (SEM, Mira3, Fig. S4) and transmission electron microscopy (TEM, Talos F200X G2, Figs. 1c and S5). As prepared ZIF-8 shows a spherical structure with an average size ~ 30 nm. After heat treatment under Ar and NH_3_. TEM shows a porous architecture within the N-pC skeleton, stemming from the evaporation of Zn accompanied by the collapse of ZIF-8 frameworks. This structure still remains porous after NH_3_ treatment. The size of the N-pC or rN-pC is ~ tens nm due to different degrees of integration between ZIF frameworks. The representative HRTEM images in Figs. 1c and S4 demonstrate that N-pC and rN-pC have similar porous and nanocrystalline graphite structure with the existence of ~ 5 layered short domains. Scanning transmission electron microscopy (STEM, Talos F200X G2, Figs. 1d and S5d) mapping of N-pC and rN-pC shows a homogenous distribution of N in the porous matrix. The N content of rN-pC is higher than N-pC (Table S3), consistent with XPS (Table S2). The higher N content and the optimized porous structure endow rN-pC with more H_2_ adsorption-active sites and surface hydrophilicity, facilitating the nucleation of Mg/MgH_2_ NPs [66].

### Hydrogen Adsorption Properties of rN-pC

**Fig. 2:**
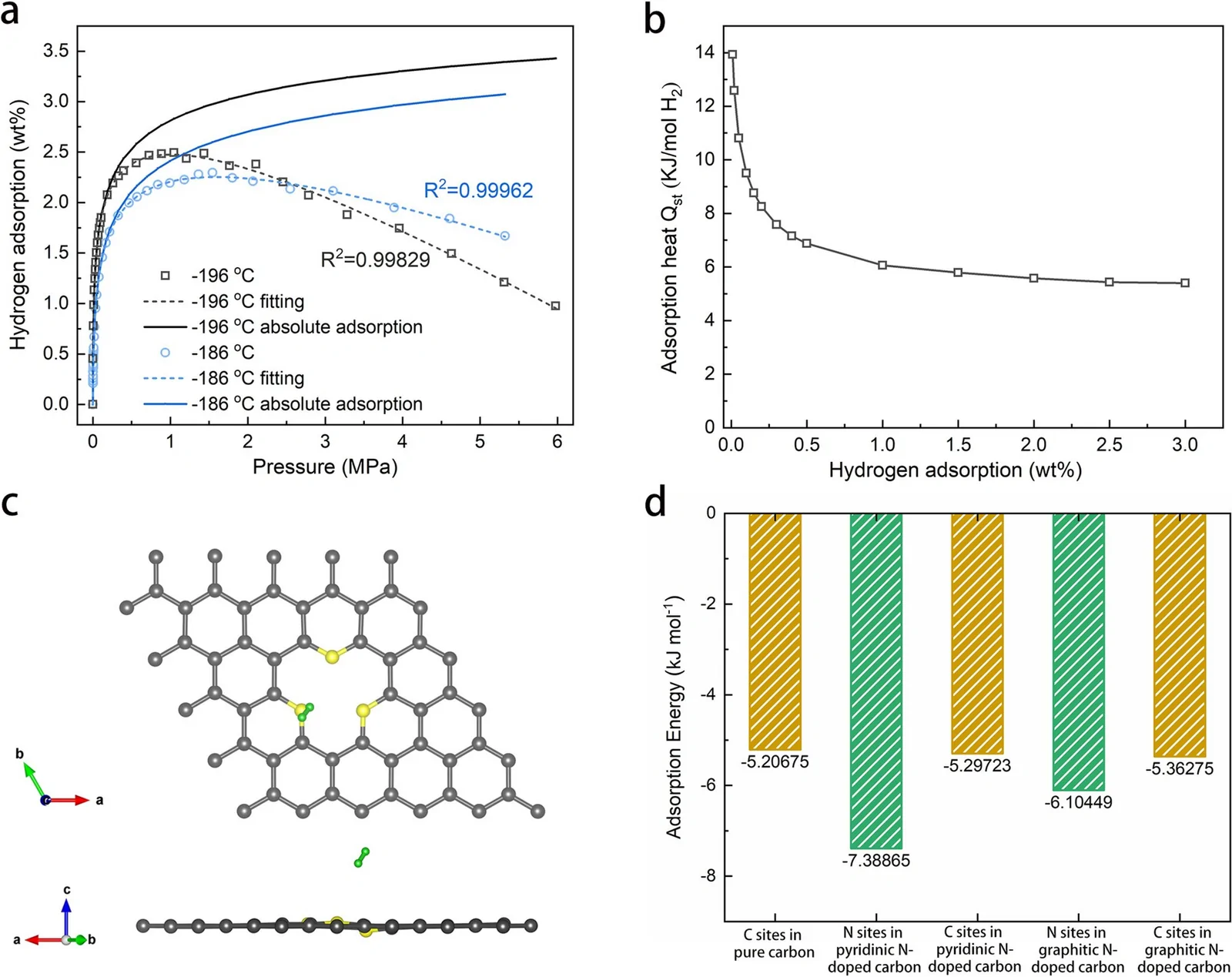
Hydrogen adsorption properties of rN-pC at cryogenic T. **a** H_2_ excess adsorption isotherms of rN-pC at -196, and -186 °C and corresponding modeling. **b** Equivalent isosteric heat of H_2_ adsorption fitting of rN-pC. **c** Top and side views of optimized geometries of H_2_ adsorbed on pyridinic N-doped graphene. **d** Adsorption energies for different substrate sites interacting with H_2_

The rN-pC has high SSA (1525.4 m^2^ g^−1^) and a certain number of micropores (0.447 mL g^−1^), suggesting potential H_2_ adsorption ability. The H_2_ storage performance of rN-pC are evaluated through high-pressure (0–6 MPa) excess H_2_ adsorption at –196 and –186 °C (Fig. 2a). These are modeled using a semi-empirical methodology (Eq. S1), then converted to absolute H_2_ uptake.

Owing to the stability of rN-pC under 750 °C treatment, the pre-activating treatment can be executed under high T > 300 °C, without any changes of structure and constituents, so that the degassing procedure can be simplified. The gravimetric H_2_ excess uptake of rN-pC increases abruptly and reaches a saturation plateau ~ 1 MPa (Fig. 2a), because N and micropores act as active adsorption sites for H_2_ adsorption. With the PSD of 0.5–30 nm in rN-pC, the H_2_ density adsorbed in mesopores is smaller than that in micropores. Therefore, a fast drop of excess adsorption is observed with increasing pressure, due to the gas density increasing with pressure faster outside than inside the pores [67]. Excess gravimetric H_2_ uptake ~ 2.5 wt% at −196 °C and ~ 0.8 Mpa, and ~ 2.3 wt% at −186 °C and ~ 1.5 MPa is detected. The saturation plateau at −186 °C shifts to a higher position compared with that at −196 °C, due to the decrease in free gas density [68]. An absolute gravimetric H_2_ uptake ~ 3.4 and ~ 3.0 wt% is recorded for rN-pC at ~ 6 MPa and −196 °C, −186 °C, respectively. The maximum volumetric H_2_ uptake ~ 17.3 g H_2_ L^−1^ is estimated considering the density functional theory (DFT, Equation S2) PV (~ 1.498 cm^3^ g^−1^) and the skeletal density (~ 2.15 g cm^−3^) of rN-pC at −196 °C.

In order to investigate the interaction between H_2_ and rN-pC, the ultralow pressure (below 0.01 MPa) H_2_ adsorption isotherms at –196 and –186 °C are shown in Fig. S6. The gravimetric H_2_ adsorptions at 0.01 MPa and −196/−186 °C are 0.9 and 0.55 wt%, respectively, meaning that rN-pC can reach a high H_2_ adsorption under a low H_2_ background pressure, indicating strong interaction compared with porous carbon [36]. The isosteric enthalpy of H_2_ adsorption (Q_st_), as derived by Langmuir fitting [69] of the ultralow pressure isotherms at −196 and −186 °C followed by a Clausius-Clapeyron calculation (Eq. S3) [70], is in Fig. 2b. The Q_st_ value at zero coverage is ~ 14 kJ mol^−1^, much higher than classical H_2_ physisorption (i.e., 1–10 kJ mol^−1^). For most standard activated carbons, with the contribution of overlapping potential fields of opposite pore walls, the zero coverage isosteric adsorption enthalpy lies in the range ~ 5–10 kJ mol^−1^, even if the binding strength of H_2_ on carbon alone is weak (~ 1–5 kJ mol^−1^) [71]. Q_st_ decreases with H_2_ uptake to ~ 6 kJ mol^−1^ at 3 wt% adsorption, indicating the existence of favorable adsorption sites. We calculate the binding energies of H_2_ on the different sites of N-doped C using DFT calculation. For H_2_ interacting with carbon materials or graphitic/pyridinic N-doped carbon materials, a (4 × 4 × 1) supercell of carbon materials doped with N was primarily simulated, and the full optimized geometries are in Figs. 2c and S7, and the interaction energies in Fig. 2d. A stronger interaction is found when H_2_ interacts with pyridinic N-doped carbon materials, with an enthalpy of 7.4 kJ mol^−1^ higher than that between H_2_ and pure carbon materials (5.2 kJ mol^−1^). Micropores with overlapping potential fields at opposite walls lead to stronger interaction between H_2_ and substrate, but this is not taken into account in our calculations (details in Experimental Section), so that the experimental values are higher than the calculated values. The improvement of H_2_ adsorption is confirmed by the density of state (DOS) and its projections for each atom and charge density difference (Figs. S8 and S9). With the contributions from N dopants, especially for pyridinic type, the DOS of substrates are closer to the Fermi level, stimulating stronger H_2_ polarization and interactions [72]. Considering the high zero coverage Q_st_ (~ 14 kJ mol^−1^) and high H_2_ adsorption (0.9 wt% at -196 °C) under ultralow relative pressure ranges (< 0.1 bar) of rN-pC, Refs. [34, 62] suggested that H_2_ adsorption of porous carbon depends on micropores, as well as its chemical composition [36, 73].

### ***Preparation and Structural Characterization of 60MgH***_***2***_***@rN-pC***

60MgH_2_@rN-pC is prepared via solution reduction of methyl magnesium chloride (CH_3_MgCl) using lithium naphthalene (LiNaph), followed by hydrogenation. The porous carbon confined Mg-based hydrogen storage composite was synthesized with robust interfaces and controllable structure through a simple and efficient method. To synthesize evenly distributed Mg NPs confined in rN-pC scaffolds, the mixture of rN-pC and CH_3_MgCl is processed under intermittent ultrasonication to promote dispersion of rN-pC in tetrahydrofuran (THF), exposing free surfaces for impregnation of Mg.

**Fig. 3:**
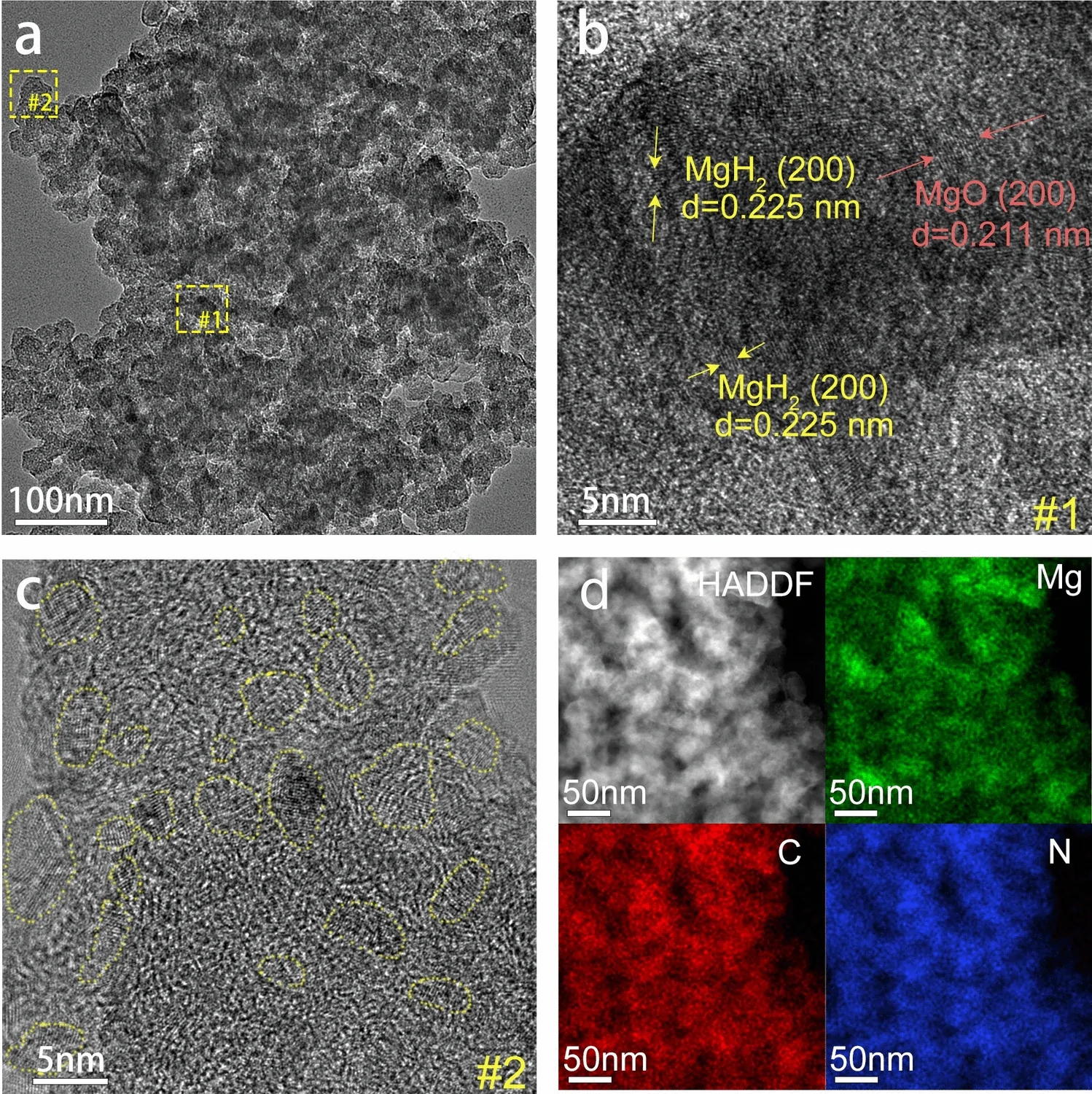
Structure and morphology characterization of 60MgH_2_@rN-pC composite. **a** The TEM and **b, c** HRTEM images of hydrogenated 60MgH_2_@rN-pC. **d** HAADF image of 60MgH_2_@rN-pC and corresponding elemental mapping for Mg, C, N

Figure 3a shows low-magnification (5.8 k) TEM images of 60MgH_2_@rN-pC. The rN-pC scaffolds maintain a porous structure, and many MgH_2_ NPs with uneven particles size homogenously distributed on the surface or in the open channel of the rN-pC (Fig. 3b, c). The absence of isolated MgH_2_ NPs indicates coupling between Mg precursor and rN-pC substrates. Correspondingly, isolated Mg NPs > 300 nm were also synthesized under the same experimental conditions, except for the addition of rN-pC (Fig. S10). The redox reaction between CH_3_MgCl and LiNaph takes place in seconds, and pre-dispersion and confinement of rN-pC is vital to obtain well-distributed Mg NPs. The microstructure of MgH_2_@rN-pC is investigated by high-resolution TEM (HRTEM, Fig. 3b). The black NPs with an average size ~ 20 nm comprise more than ten MgH_2_ grains with different orientations. The interplanar spacing ~ 0.225 nm corresponds to the (200) planes of MgH_2_, while the interplanar spacing ~ 0.211 nm is assigned to the (200) planes of MgO [74]. The active MgH_2_ NPs are oxidized by the residual moisture in the THF solvent and during the preparation and transportation before TEM measurement, consistent with the diffraction ring of MgO in the SAED pattern of Fig. S11. Figure 3c confirms that MgH_2_ NPs < 5 nm are confined in the channel of rN-pC, as reported in literature [19], with outstanding H_2_ thermodynamic and kinetic performances. Besides, the 60MgH_2_@rN-pC heterostructure still maintains its initial structure and shows no obvious agglomeration of MgH_2_ after 10 cycles (Fig. S12). Fourier transform infrared (FTIR) spectra of rN-pC, pure MgH_2_ and 60MgH_2_@rN-pC are also consistent with the introduction of MgH_2_ NPs (Fig. S13). STEM mapping in Fig. 3d indicates homogeneous distribution of Mg, C, N in the MgH_2_@rN-pC, in agreement with TEM. The BET and PSD analyses of rN-pC and 60MgH_2_@rN-pC based on DFT are in shown in Fig. S14. BET tests give a 60MgH_2_@rN-pC SSA ~ 636.5 m^2^ g^−1^, much lower than pristine rN-pC (1525.4 m^2^ g^−1^). The rN-pC PSD plot < 2.5 nm has a peak ~ 0.59 nm, not present for 60MgH_2_@rN-pC. This has 3 humps ~ 0.75, 1.3, and 1.6 nm, consistent with rN-pC, but with decreased average density. MgH_2_ NPs are confined in the pore channel or cover the pore entrance, reducing SSA and the corresponding rN-pC pore size.

### ***H***_***2***_*** Storage Properties of 60MgH***_***2***_***@rN-pC***

**Fig. 4:**
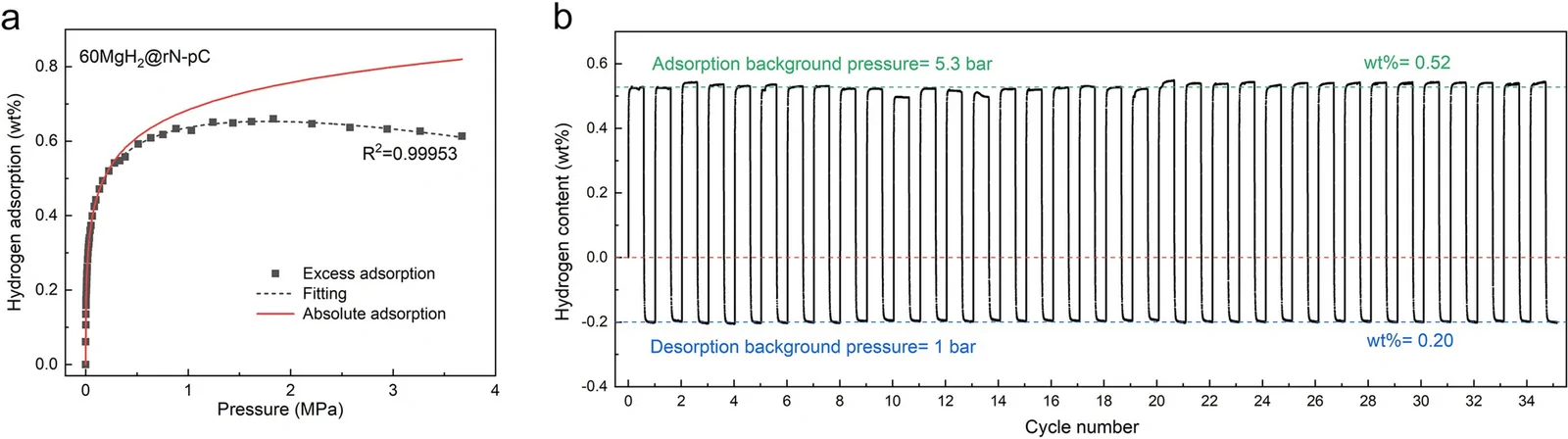
Hydrogen adsorption properties of 60MgH_2_@rN-pC composite at cryogenic T. **a** Experimental and modeled H_2_ excess adsorption isotherms of 60MgH_2_@rN-pC at -196 °C. **b** Cycling H_2_ adsorption/desorption of 60MgH_2_@rN-pC at -196 °C

The H_2_ hydrogen storage capacity of 60MgH_2_@rN-pC at cryogenic (–196 and –186 °C) and high T up to 350 °C was tested. The data about physical adsorption of the composites were all collected in the state that Mg was fully hydrogenated. First, the gravimetric H_2_ excess uptake of 60MgH_2_@rN-pC is measured at –196 °C. As for Fig. 4a, the H_2_ excess adsorption content decreases to 0.66 wt% (2.5 wt% for rN-pC) and the adsorption saturation pressure shifts to higher value (~ 1 Mpa for rN-pC and ~ 1.8 Mpa for 60MgH_2_@rN-pC). The isosteric enthalpy of H_2_ adsorption (Q_st_) is calculated by Langmuir fitting of the ultralow pressure isotherms at –196 and –186 °C (Fig. S15). The zero coverage Q_st_ is estimated to be ~ 10.6 kJ mol^−1^, in the range reported in literatures for H_2_ physisorption [75]. A possible reason for the decrease of H_2_ adsorption capacity and lowering of zero coverage Q_st_ could be related to the filling and coverage of pores by MgH_2_ NPs, resulting in the reduction of effective contacts between H_2_ and C, as well as active N sites. However, micropores in the carbon scaffold are less accessible for Mg precursors [76], which means that most nucleates on the mesopores and surfaces and partial micropores are retained. From the semi-empirical calculations described in Eq. S1, the H_2_ adsorption density is ~ 0.045 g cm^−3^ (Table S4, 0.026 g cm^−3^ for rN-pC), indicating that it is possible to obtain an improved volumetric H_2_ storage system. The H_2_ cycling ad/desorption performance of 60MgH_2_@rN-pC is measured at -196 °C. As shown in Fig. 4b, H_2_ adsorption is totally reversible, with no hysteresis. The excellent cycling stability indicates that 60MgH_2_@rN-pC maintains a stable porous structure and MgH_2_ NPs are well bound to the rN-pC framework, without significant detachment under cryogenic T. This study shows the first realization of nanoconfined Mg-based system with adsorption-active scaffolds (Table 1).

**Table 1:** Hydrogen storage performance parameters of different Mg-carbon material nanocomposites

Carbon material	MgH_2_ content (wt%)	Synthetic method	Onset desorption temperature of MgH_2_ (°C)	Kinetic performance of MgH_2_	H_2_ pysi-/chemical sorption (wt%)	References
graphite	90	mechanical milling	280	De: 6.5 wt%/ 300 °C/30 kPa/ 20 min	–/6.5	[85]
GNS	95	mechanical milling	300	De: 1.3 wt%/ 325 °C/-/20 min	–/6.0	[86]
CNC	90	mechanical milling	245	De: 5.3 wt%/ 325 °C/-/10 min	–/5.4	[38]
CNT	90	mechanical milling	247	De: 6.0 wt%/ 300 °C/vacuum/ 3 h	–/6.5	[87]
graphene	60	nanoconfinement	250	De: 4.5 wt%/ 320 °C/1 kPa/80 min	–/4.5	[88]
CMK3	37.5	nanoconfinement	50	De: 2.1 wt%/ 300 °C/-/8 h	–/2.25	[84]
carbon aerogel	18.2	nanoconfinement	200	De: 0.9 wt%/ 300 °C/-/6 h	–/1.4	[25]
graphene	75	nanoconfinement	200	De: 2.6 wt%/ 200 °C/1 kPa/ 200 min	–/5.7	[24]
BCNTs	75	nanoconfinement	210	De: 5.8 wt%/300 °C/2 kPa/30 min	–/5.84	[89]
pCNF	60	nanoconfinement	230	De: 1.6 wt%/250 °C/1 kPa/120 min	–/4.0	[90]
MOF-derived carbon	–	–	–	–	3.25/–	[91]
MgH_2_@optimized carbon	60	nanoconfinement	175	De: 3.3 wt%/300 °C/10 kPa/15 min	0.7/3.6	This work

**Fig. 5:**
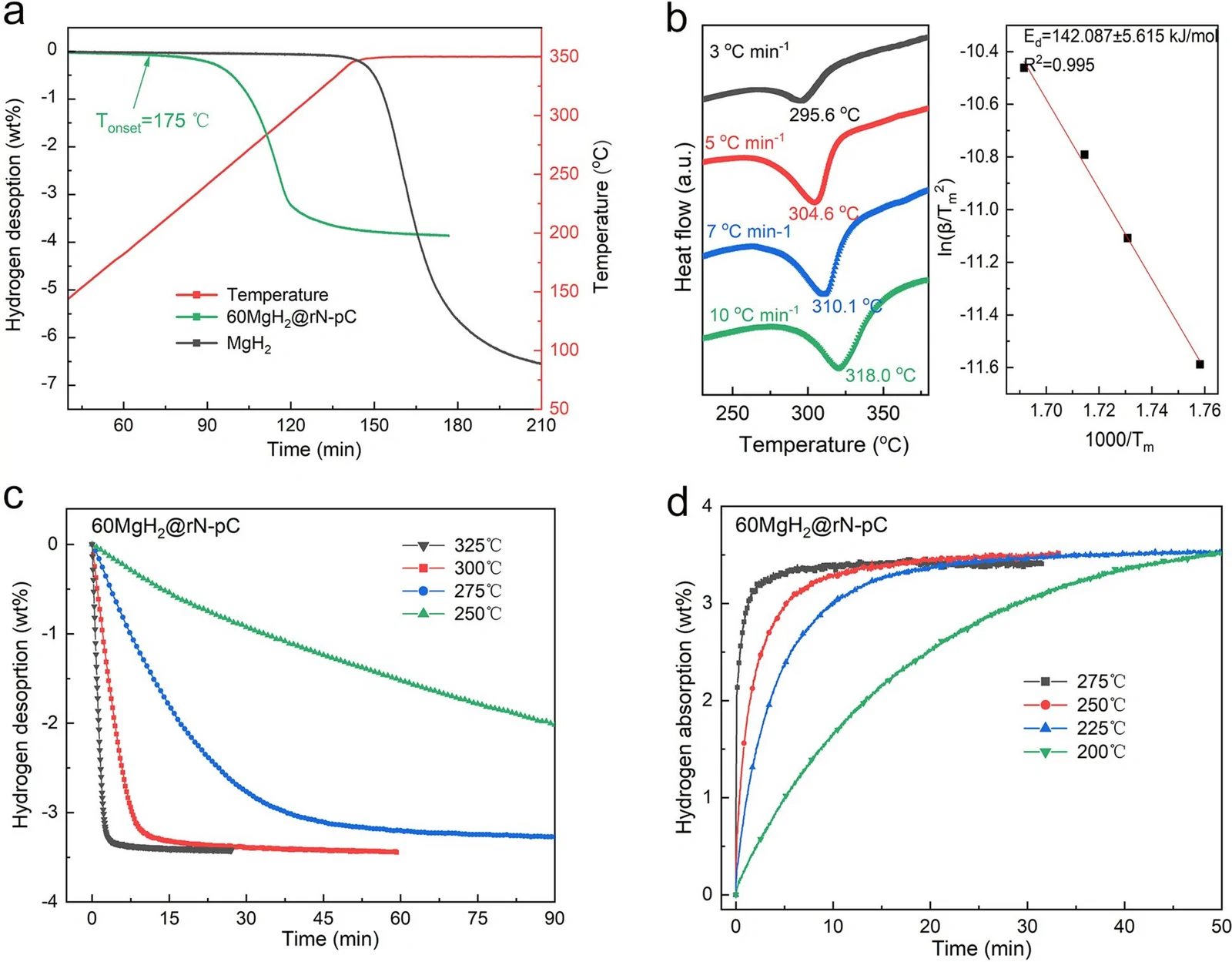
Hydrogen absorption and desorption properties of 60MgH_2_@rN-pC composite at elevated T. **a** TPD of pure MgH_2_ and 60MgH_2_@rN-pC. **b** DSC and corresponding Kissinger’s plots of 60MgH_2_@rN-pC. **c** dehydrogenation and **d** re-hydrogenation curves of 60MgH_2_@rN-pC at different T

We then study the high T (0–350 °C) H_2_ storage properties of the pure MgH_2_ and 60MgH_2_@rN-pC by T Programmed Desorption (TPD) analysis of 60MgH_2_@rN-pC in comparison with pure MgH_2_ is in Fig. 5a. The samples for TPD analysis were obtained after initial hydrogenation. Pure MgH_2_ starts to desorb H_2_ at ~ 299 °C with a final capacity ~ 6.8 wt%. The T_onset_ of 60MgH_2_@rN-pC reduces to 175 °C, with a final capacity of 3.85 wt%. A small amount of Mg/MgH_2_ reacted irreversibly with impurities in the chemicals to form MgO, leading to the capacity loss. ~ 3 wt% H_2_ is released from 60MgH_2_@rN-pC before 300 °C, while pure MgH_2_ starts to decompose at the same T. Differential scanning calorimetry (DSC) experiments are then performed at different heating rates ~ 3, 5, 7, 10 °C min^−1^ to further demonstrate the improvement of desorption properties (Fig. 5b). The peak desorption T of 60MgH_2_@rN-pC is 295.6 °C at the heating rate of 3 °C min^−1^, 82.7 °C lower than that pure MgH_2_ (378.3 °C) (Fig. S16). The apparent activation energy of desorption (E_a_) is calculated from the Kissinger’s equation (Eq. S4) [77]. Figures 5b and S16 show a linear relation between ln(β/T_p_^2^) and 1000/T_p_ (β represents the heating rate, Tp refers to the peak temperature) for 60MgH_2_@rN-pC and MgH_2_. E_a_ of 60MgH_2_@rN-pC is fitted to ~ 142.1 ± 5.6 kJ mol^−1^ H_2_, much lower than pure MgH_2_ (189.2 ± 12.8 kJ mol^−1^ H_2_), consistent with the calculation using the Johnson–Mehl–Avrami–Kolmogorov (JMAK) and Arrhenius equation [78] (Fig. S17, 148.2 ± 3.3 kJ mol^−1^ H_2_ and 181.6 ± 0.8 kJ mol^−1^ H_2_ for 60MgH_2_@rN-pC and pure MgH_2_, respectively). The high desorption activation energy of pure MgH_2_ comes from its surface structure with good crystallinity, as shown in Fig. S10, which is close to the theoretical calculation results [79, 80]. For the fitting of 60MgH_2_/rN-pC desorption curves, the parameter n was determined as ~ 1.1, which means that the reaction is controlled by particle surface reaction (first-order reaction) and the MgH_2_/rN-pC interfaces serve as the active nucleation sites for Mg [81, 82]. In contrast, for the fitting of MgH_2_ desorption curves, the parameter n was determined to be ~ 3.5, indicating that the inner and outer regions of hydrides are dehydrogenated simultaneously driven by high temperature and Mg nuclei form randomly inside the bulk [83]. The isothermal hydrogenation/dehydrogenation kinetics of 60MgH_2_@rN-pC and MgH_2_ are then measured. Figure 5c plots the isothermal desorption curves of 60MgH_2_@rN-pC at different T. 60MgH_2_@rN-pC releases 0.93, 2.76, 3.38, and 3.41 wt% H_2_ at 250, 275, 300, and 325 °C within 30 min. At 325 °C, 60MgH_2_@rN-pC can fully decompose within 5 min, while MgH_2_ takes > 2 h (Fig. S18(a)). 60MgH_2_@rN-pC releases almost all H_2_ within 5 h at 250 °C. Even at a lower T = 225 °C, the composite can still desorb 2 wt% H_2_ within 8 h, corresponding to 58.8% of the maximum H_2_ storage capacity (Fig. S19). In contrast, pure MgH_2_ can only release < 0.5 wt% H_2_ at 300 °C within 3 h. Such an improved dehydrogenation performance confirms that the rN-pC scaffolds have a positive effect on MgH_2_ dehydrogenation. The isothermal hydrogenation curves of dehydrogenated 60MgH_2_@rN-pC and MgH_2_ are recorded at different T for 3.2 MPa H_2_ pressure (Figs. 5d and S18b). The dehydrogenated reference sample can only absorb ~ 0.72 and 4.71 wt% at 200 and 250 °C, corresponding to 10.3% and 67.3% of its maximum H_2_ storage capacity, respectively. The dehydrogenated 60MgH_2_@rN-pC has a fast absorption kinetics, as it can absorb 3.5 wt% H_2_ at 200 °C with 50 min. Compared with Mg-carbon material composites reported in literatures, 60MgH_2_@rN-pC shows relatively lower T_onset_ and enhanced desorption kinetics (Table 1). Nanoconfinement strategy has shown great ability to improve the hydrogen storage performances of Mg-based hydrogen storage materials, especially for the initial destabilization at relatively low temperature due to the interfacial catalysis [24, 84]. Besides, in this study, we investigate hydrogen adsorption ability of the supporting materials, which has the potential to address the hydrogen supply gap of Mg-based hydrogen storage materials at near-ambient temperatures. Pressure-Composition-T (PCT) absorption curves are recorded at 320, 340, and 360 °C (Fig. S20a). Only one sorption pressure plateau can be observed for each isotherm, suggesting a single-step transition between Mg and MgH_2_ phases during H_2_ uptake. According to the van’t Hoff plot (Eq. S5) [92], and fitting in Fig. S20b, the hydrogenation enthalpy for 60MgH_2_@rN-pC is determined to be ~ 67.98 ± 0.66 kJ mol^−1^ H_2_, much lower than MgH_2_ reported in the literatures (~ 75 kJ mol^−1^ H_2_) [14], confirming that MgH_2_ NPs are confined in rN-pC scaffolds, with improved thermodynamics. In order to evaluate the cycling stability of 60MgH_2_@rN-pC during the thermal de/hydrogenation processes, 10 isothermal hydrogenation/dehydrogenation cycles are performed at 275 °C. Figure S21 shows that 60MgH_2_@rN-pC has a stable H_2_ sorption capacity for 10 cycles (from 3.52 wt% to 3.47 wt% for absorption and from 3.24 wt% to 3.17 wt% for desorption). Because the dehydrogenation of pure MgH_2_ cannot occur at 275 °C (Fig. S18a), its cycling properties are tested at a higher T = 350 °C. Pure MgH_2_ exhibits severe degradation after 10 cycles, with desorption capacity reducing from 7.3 wt% to 5.9 wt% (Fig. S22), and the kinetic performance of pure MgH_2_ decays significantly after 15 cycles (Fig. S23). Reversible hydrogen absorption/desorption cycling profiles of the pure MgH_2_ at 350 °C for 25 cycles and 60MgH_2_@rN-pC at 300 °C for 30 cycles also show the good stability of 60MgH_2_@rN-pC (Fig. S24), which can be ascribed to the confinement of rN-pC on MgH_2_ and robust interfacial contact between host and supporting materials.

### ***Catalytic Mechanism of the 60MgH***_***2***_***@rN-pC***

**Fig. 6:**
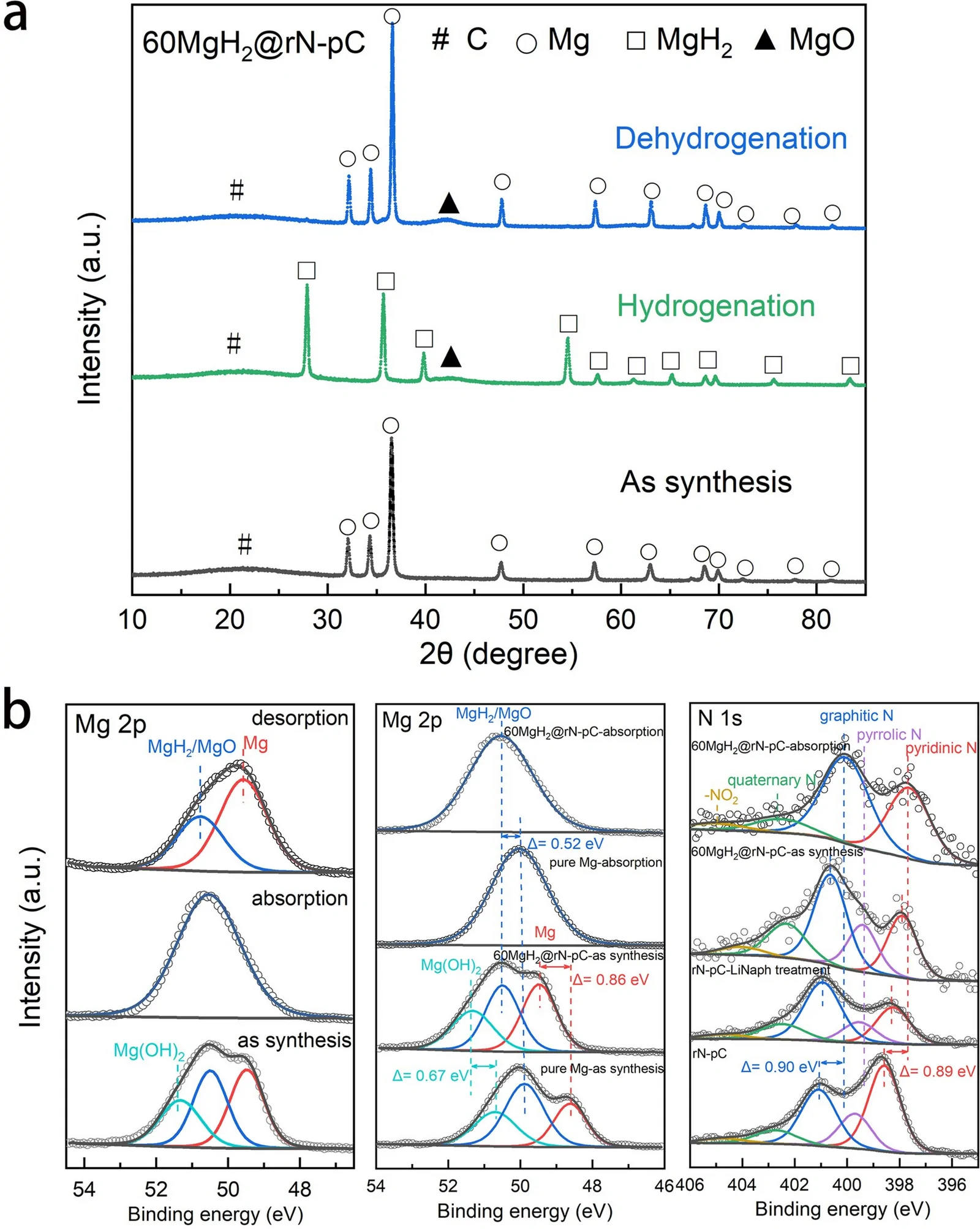
Phase and electron structure change of 60MgH_2_@rN-pC composite during the absorption and desorption processes. **a** XRD patterns of 60MgH_2_@rN-pC. **b** XPS spectra of Mg 2*p* for 60MgH_2_@rN-pC (left), Mg 2*p* for pure MgH_2_ and 60MgH_2_@rN-pC (middle), and N 1*s* for rN-pC and 60MgH_2_@rN-pC (right)

In order to investigate the mechanisms leading to improved sorption performance, we characterize the phase transformation during the thermal de/re-hydrogenation (Fig. 6a). The XRD curves of as-synthesized samples show diffraction peaks of Mg (PDF#00–004-0770) and a broad peak of C, indicating the successful loading of Mg NPs. After hydrogenation, the main peaks of MgH_2_ (PDF#00–012-0697) derive from the absorption of Mg NPs [93]. A MgO (PDF#00–035-1184) peak appears ~ 42.7°, indicating amorphous impurities [94], which might react with Mg NPs during H_2_ absorption. In the H_2_ desorption process, MgH_2_ is transformed to Mg and the peak assigned to MgO shows no change. In the whole process of H_2_ absorption and desorption, there is no new phase generated besides Mg, MgH_2_, MgO, with absence of additional catalytic phases.

H_2_ isothermal absorption tests suggests that the thermodynamic destabilization of MgH_2_ could be attributed to the “nano-size effect” of MgH_2_ NPs, usually considered a key factor for thermodynamic improvement [19], since the typical crystallite sizes of MgH_2_ are < 10 nm. Meanwhile, nanoconfined MgH_2_ NPs have a close contact with the rN-pC scaffold, introducing extra interfacial energy into the system. For MgH_2_ loaded in CMK3 composites, Jia et al. calculated that the charge transfer from MgH_2_ to CMK3 plays an important role in weakening Mg-H bonds [84].

Benefitting from the nondestructive synthetic method, the robust interfacial structure between MgH_2_ and rN-pC was achieved and the electron transfer in the interfaces was revealed by experimental characterizations. We analyzed XPS of samples at different stages of synthesis, absorption and desorption to investigate the effect of interfaces (Fig. 6b). The three peaks at ~ 49.5, 50.5, 51.3 eV for as-synthesized MgH_2_@rN-pC belong to Mg, MgO, and amorphous Mg(OH)_2_, respectively [24]. The peaks of Mg and Mg(OH)_2_ disappear due to the complete hydrogenation of Mg and decomposition of Mg(OH)_2_, consistent with the XRD results of Fig. 6a. The Mg peaks reappear in the XRD patterns of samples after desorption. To evaluate the interaction between MgH_2_ and rN-pC in the 60MgH_2_@rN-pC composite, we synthesized pure MgH_2_ and rN-pC using exactly the same methods, which were characterized as the “initial states” of the components. Then, the XPS signals of the 60MgH_2_@rN-pC composite were collected to obtain the “final states” of the components. We conclude that the signal changes can be used to deduce the catalytic effects of rN-pC, which has been widely adopted in the literatures [95–97]. Compared with pure Mg, the Mg and MgH_2_ peaks shift to higher binding energies in MgH_2_@rN-pC. Correspondingly, the N peaks of MgH_2_@rN-pC shift to lower binding energies than rN-pC (Fig. 6b). The electronegativity of N (3.07) is much higher than Mg (1.30) and C (2.54) [98]. The changes of peak positions indicate that charges transfer from MgH_2_/Mg to rN-pC, consistent with the calculations of Ref. [99]. Combined with the reduced NPs size of Mg/MgH_2_ and increased electronegativity of substrates, the interfacial effects impact the absorption/desorption properties of MgH_2_. Moreover, XPS spectra of Mg 2*p* and N 1*s* in the 60MgH_2_@rN-pC composite after 10 re/dehydrogenation cycles show the same changing tendency of peak positions as that appears in the initial cycling state, indicating that the charge transfer from MgH_2_/Mg to rN-pC is stable, as well as the interfacial structures (Fig. S25).

**Fig. 7:**
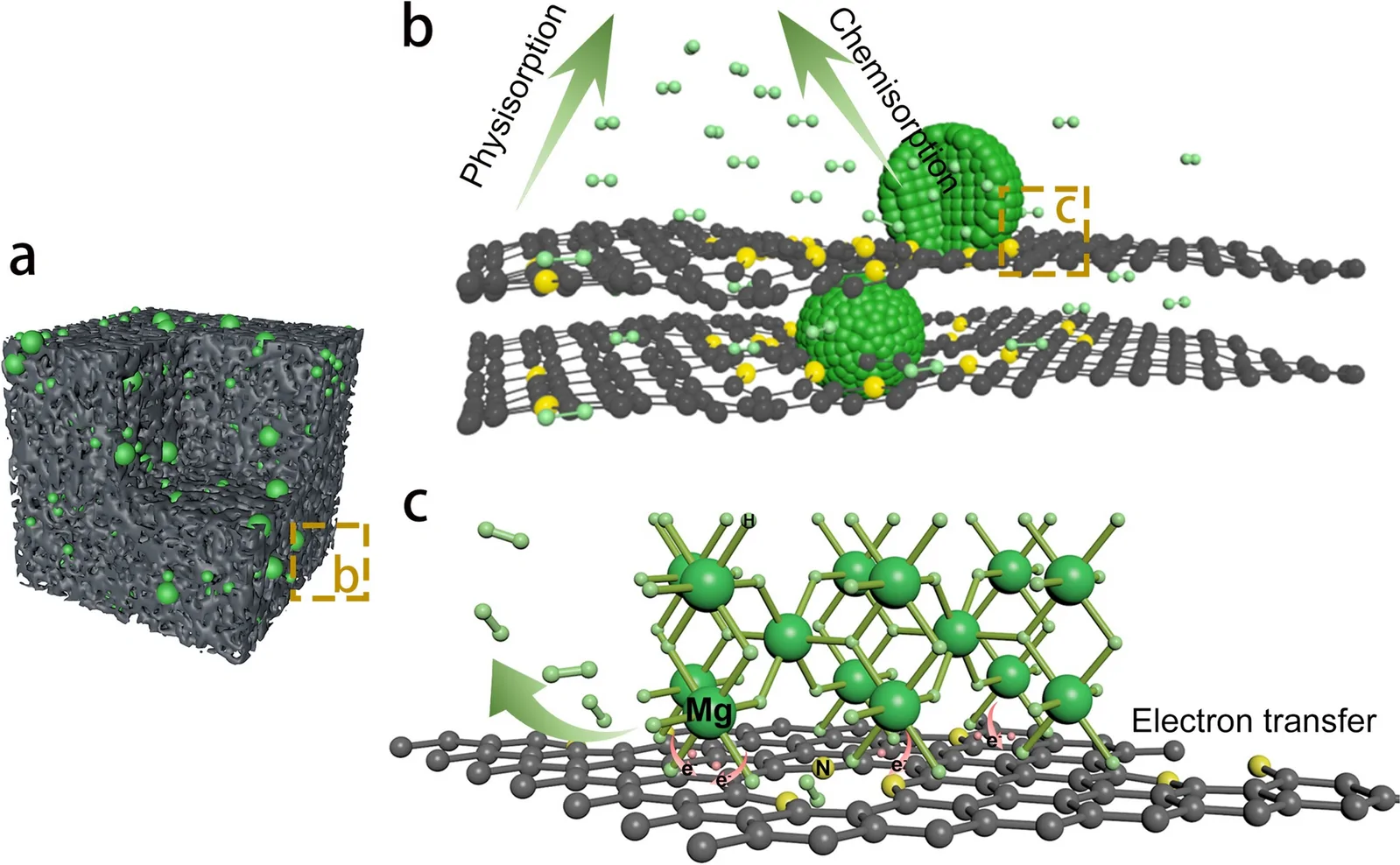
Illustration of hybrid physical and chemical H_2_ sorption in MgH_2_@rN-pC composite

Thus, our MgH_2_ NPs are confined in a porous carbon matrix possessing H_2_ adsorption capacity, besides benefiting from the catalytic activity and nanoconfinement effects of rN-pC (Fig. 7). MgH_2_@rN-pC has both physical and chemical sorption properties, lowered H_2_ desorption T than MgH_2_, and improved cycling stability. As shown in Fig. S26 and its corresponding explanation, at RT H_2_ adsorbed in rN-pC scaffolds desorbs spontaneously and ~ 0.6 wt% is released firstly. Subsequently, when the system is further heated, nano-MgH_2_ in the composite continues releasing ~ 4.2 wt% H_2_ in a wide T range (–196−325 °C). The maximum volumetric H_2_ uptake is calculated to be ~ 43.5 g H_2_ L^−1^, considering the DFT PV (~ 0.440 cm^3^ g^−1^), skeletal density (~ 1.904 g cm^−3^) of rN-pC. The exhaust heat from working Fuel Cell supplied by physical desorbed H_2_ can be used as the heat resource for MgH_2_ decomposition in the second stage. Besides, MgH_2_@rN-pC can be compacted and remain a high volumetric capacity (33.4 g H_2_ L^−1^, Figs. S27 and S28). The PSD can be further optimized to achieve higher H_2_ storage density.

## Conclusions

We presented a strategy to synthesize rN-pC as the supporting scaffold for nanoconfinement of Mg/MgH_2_ NPs. Benefitting from the porous scaffolds and confined MgH_2_ NPs, MgH_2_@rN-pC shows promising H_2_ storage performances with both physical adsorption and chemisorption properties. It can adsorb 0.62 wt% H_2_ at -196 °C and release H_2_ spontaneously with increasing T. MgH_2_ NPs start to decompose at 175 °C to offer ~ 3.59 wt% H_2_ continuously with a faster desorption kinetics. The Mg NPs thermodynamics can be also improved with a MgH_2_ formation enthalpy ~ 68 kJ mol^−1^ H_2_, mainly due to nanosize effects. For applications, the composite can be compacted to pellets with a volumetric H_2_ density as high as 33.4 g L^−1^ at 500 MPa. Thanks to a strong coupling between MgH_2_ NPs and rN-pC, in situ formed heterogeneous interfaces induce the charge transfer from Mg/MgH_2_ to rN-pC, thereby weakening the Mg-H bonds. MgH_2_@rN-pC displays an achieved cycling stability at both cryogenic (-196 °C) and elevated (275 °C) T. Our work offers a new approach for the design of H_2_ storage systems with higher H_2_ content through the nanoconfinement of MgH_2_ using H_2_ adsorbents. To develop materials that meet the US Department of Energy’s (DOE’s) targets [100], scaffolds are needed to improve H_2_ adsorption capacity at RT and to catalyze MgH_2_ to release H_2_ at ambient T.

## Supplementary Information

Below is the link to the electronic supplementary material.

Supplementary file1 (DOCX 30491 KB)
