# Supplementary material for: Achieving Wide-Temperature-Range Physical and Chemical Hydrogen Sorption in a Structural Optimized Mg/N-Doped Porous Carbon Nanocomposite
Source: Nanomicro Lett. 2026 Jan 2;18:94. doi: 10.1007/s40820-025-01931-w (PMC12757507; doi:10.1007/s40820-025-01931-w)
Supplement: Supplementary file 1 — Supplementary file1 (DOCX 30491 KB) [file 40820_2025_1931_MOESM1_ESM.docx]

Supporting Information for

**Achieving Wide Temperature Range Physi- and Chemical Hydrogen Sorption in a Structural Optimized Mg/N-Doped Porous Carbon Nanocomposite**

Yinghui Li ^1, 2^, Li Ren^1, 2^, Zi Li^1, 2^, Yingying Yao^1, 2^, Xi Lin^1^, Wenjiang Ding^1, 2^, Andrea C. Ferrari^3, *^, Jianxin Zou^1, 2, 3, *^

^1^Shanghai Key Laboratory of Hydrogen Science & Center of Hydrogen Science, Shanghai Jiao Tong University, Shanghai 200240, P. R. China

^2^National Engineering Research Center of Light Alloys Net Forming & State Key Laboratory of Metal Matrix Composites, Shanghai Jiao Tong University, Shanghai, 200240, P. R. China

^3^Cambridge Graphene Centre, University of Cambridge, Cambridge, CB3 0FA, U. K.

*Corresponding authors. E-mail: [acf26@eng.cam.ac.uk](mailto:acf26@eng.cam.ac.uk) (Andrea C. Ferrari); [zoujx@sjtu.edu.cn](mailto:zoujx@sjtu.edu.cn) (Jianxin Zou)

**Supplementary** **Figures**


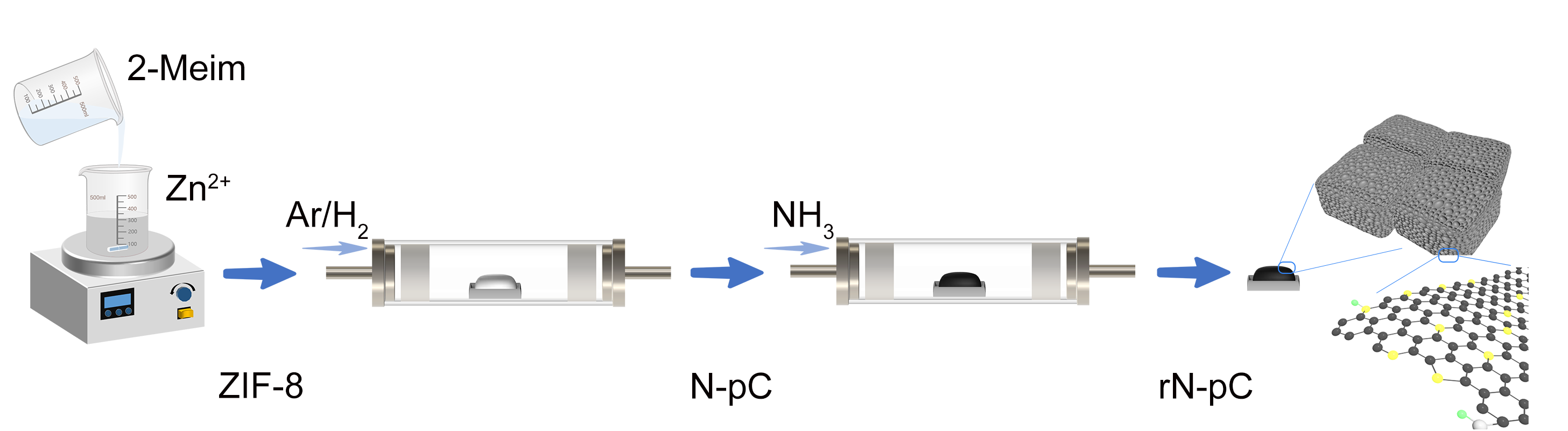


**Fig. S1** Schematic illustration of rN-pC preparation


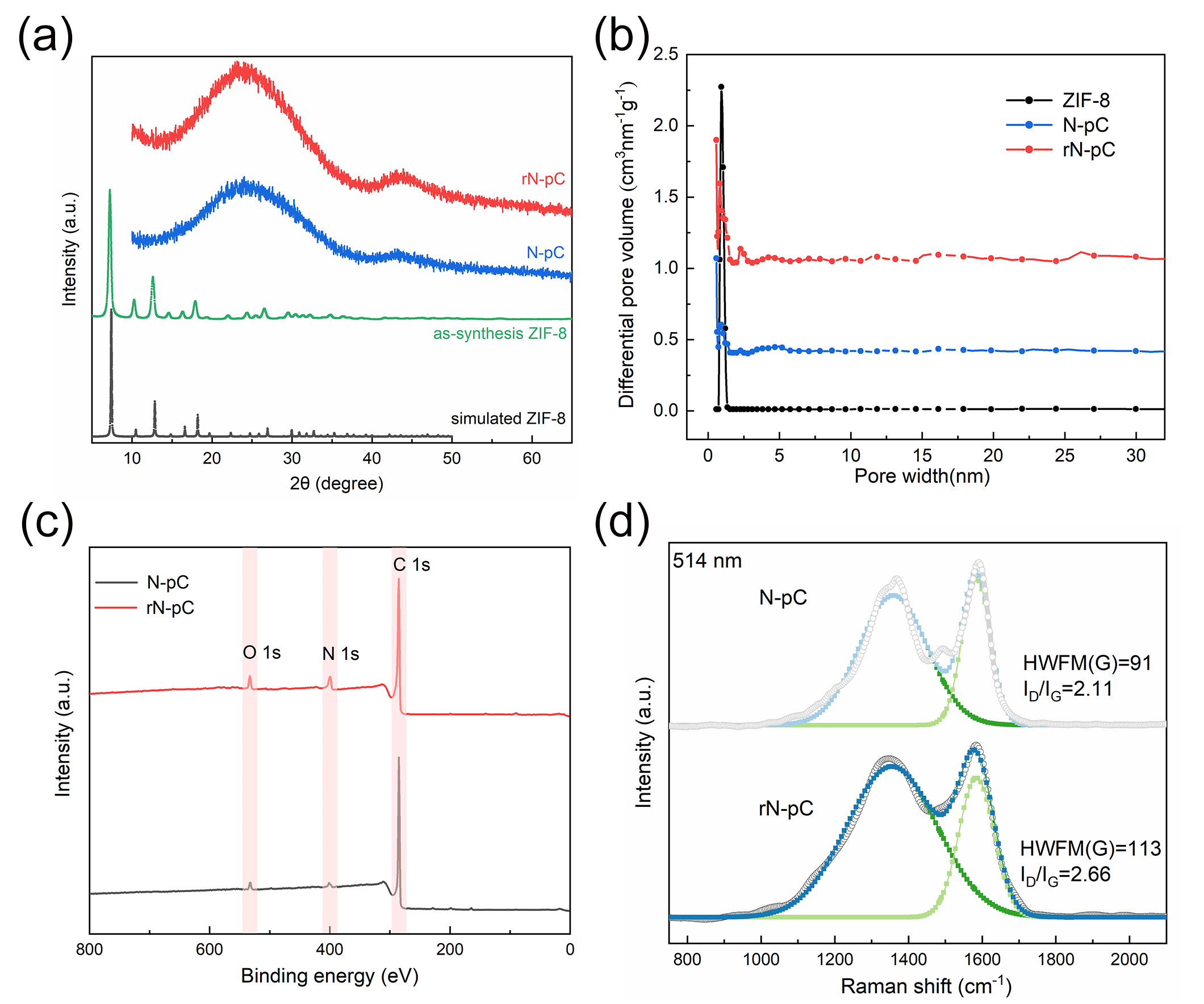


**Fig. S2** **a** XRD patterns of ZIF-8 (simulated), as-synthesized ZIF-8, N-pC and rN-pC. **b** Pore-size distribution of ZIF-8, N-pC and rN-pC. **c** Survey XPS spectra of N-pC and rN-pC and **d** Raman spectra of N-pC and rN-pC under excitation of 514 nm laser


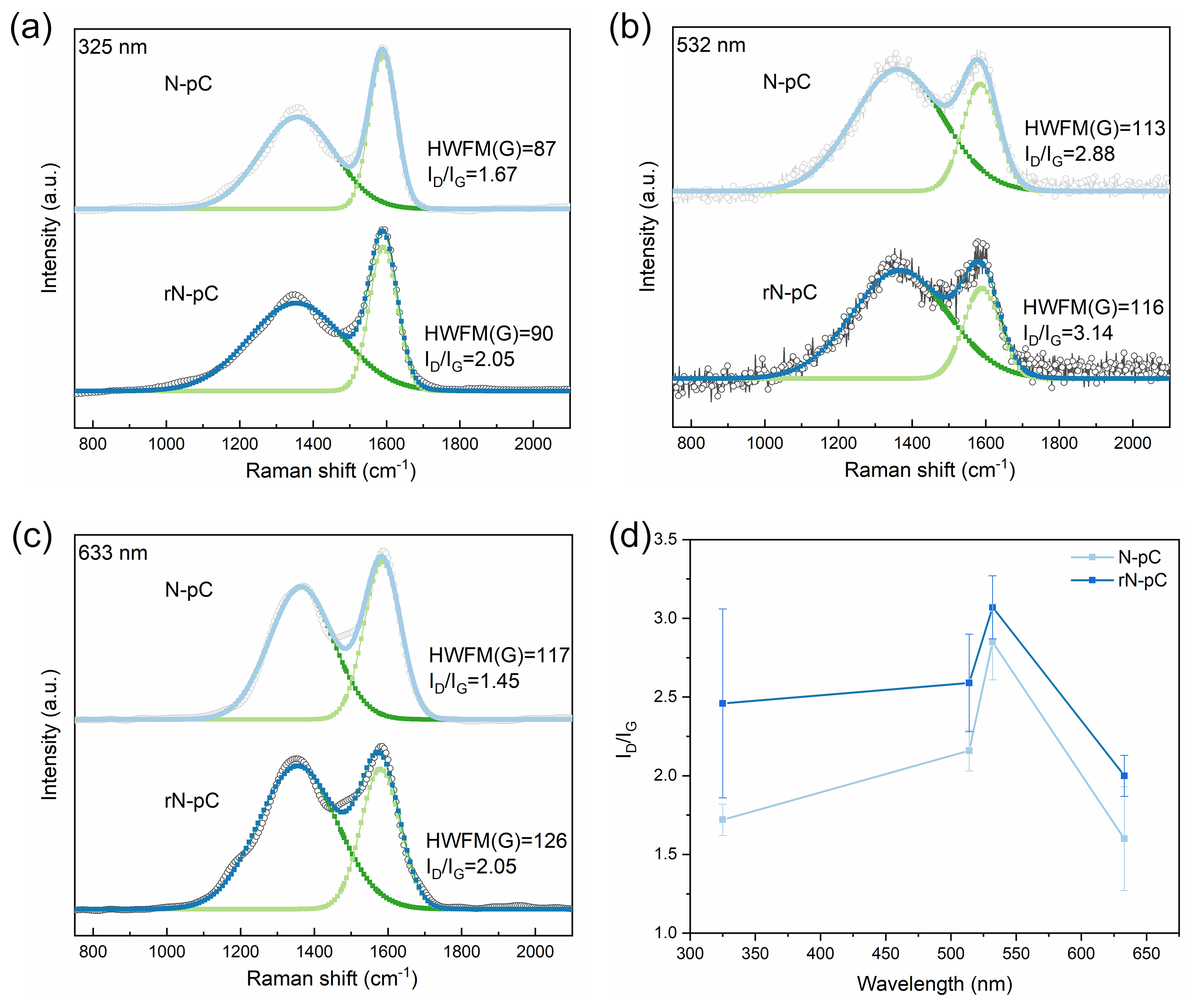


**Fig. S3** Raman spectra of N-pC and rN-pC under excitation of **a** 325nm, **b** 532nm, **c** 633nm lasers, and **d** I(D)/I(G) (the ratio of peak areas) dispersion with the change of excitation wavelengths with error bars


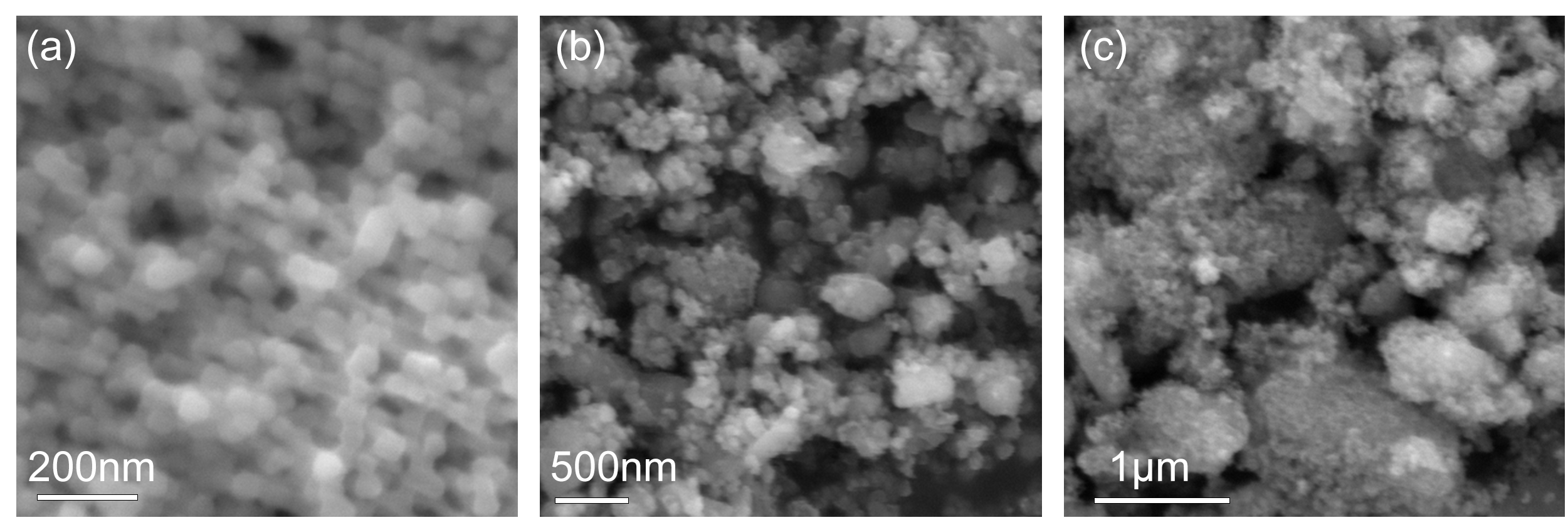


**Fig. S4** SEM images of **a** ZIF-8, **b** N-pC and **c** rN-pC


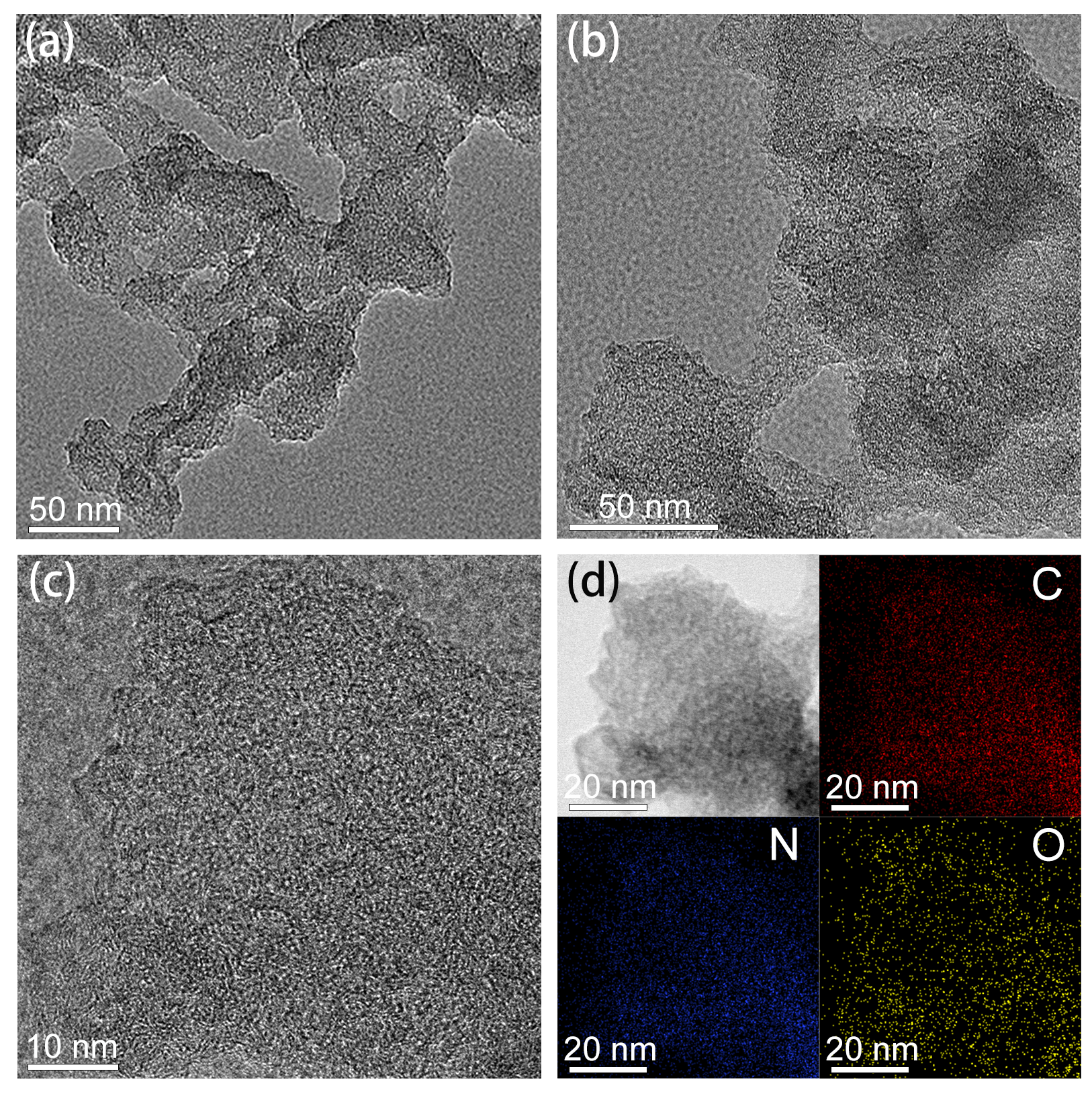


**Fig. S5** Typical TEM images of **a** rN-pC and **b, c** N-pC at different scales, and **d** representative BF image as well as corresponding EDS elemental mapping results of N-pC


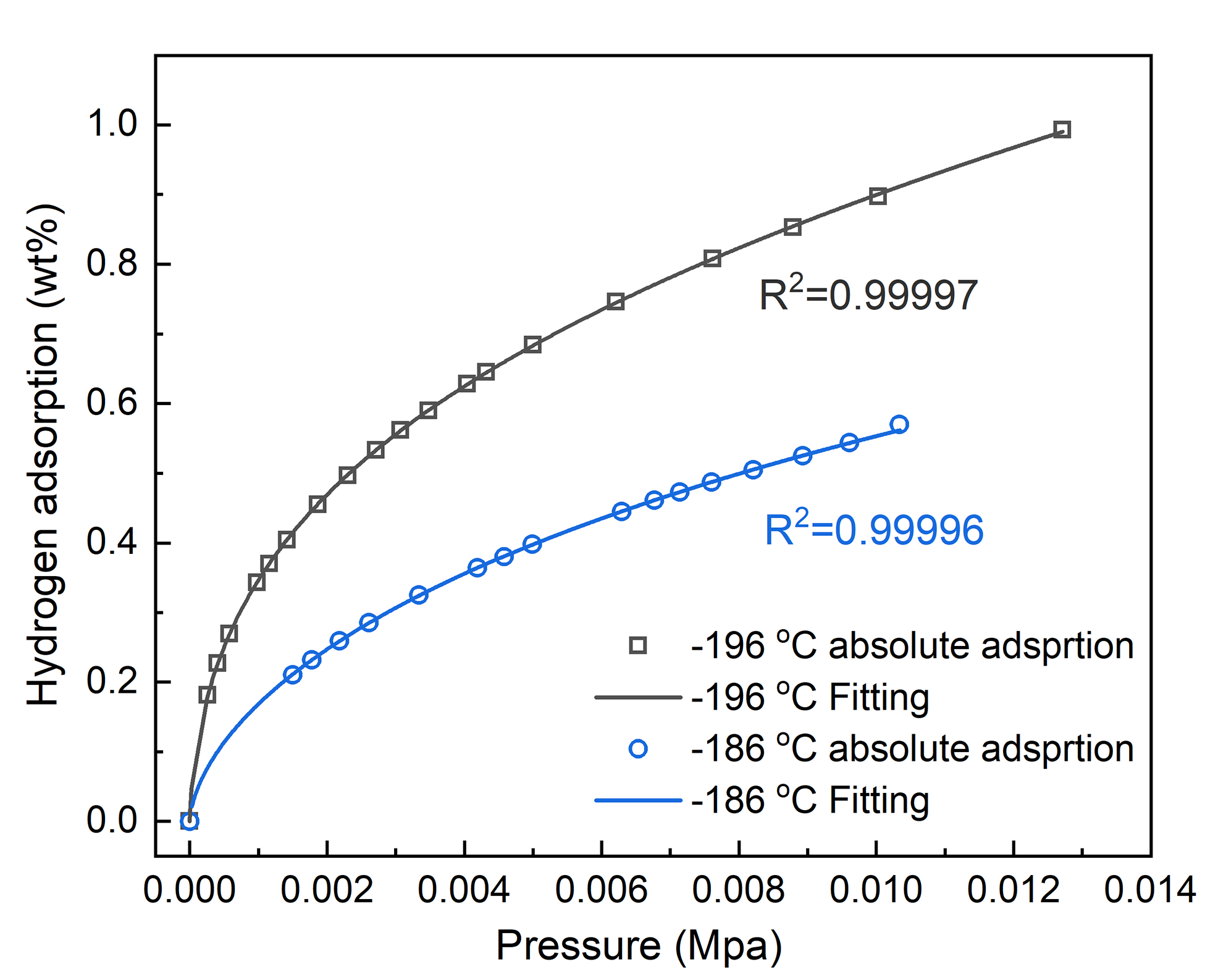


**Fig. S6** Experimental H_2_ adsorption and fitting of rN-pC at 196 and -186 ^o^C under ultralow pressure


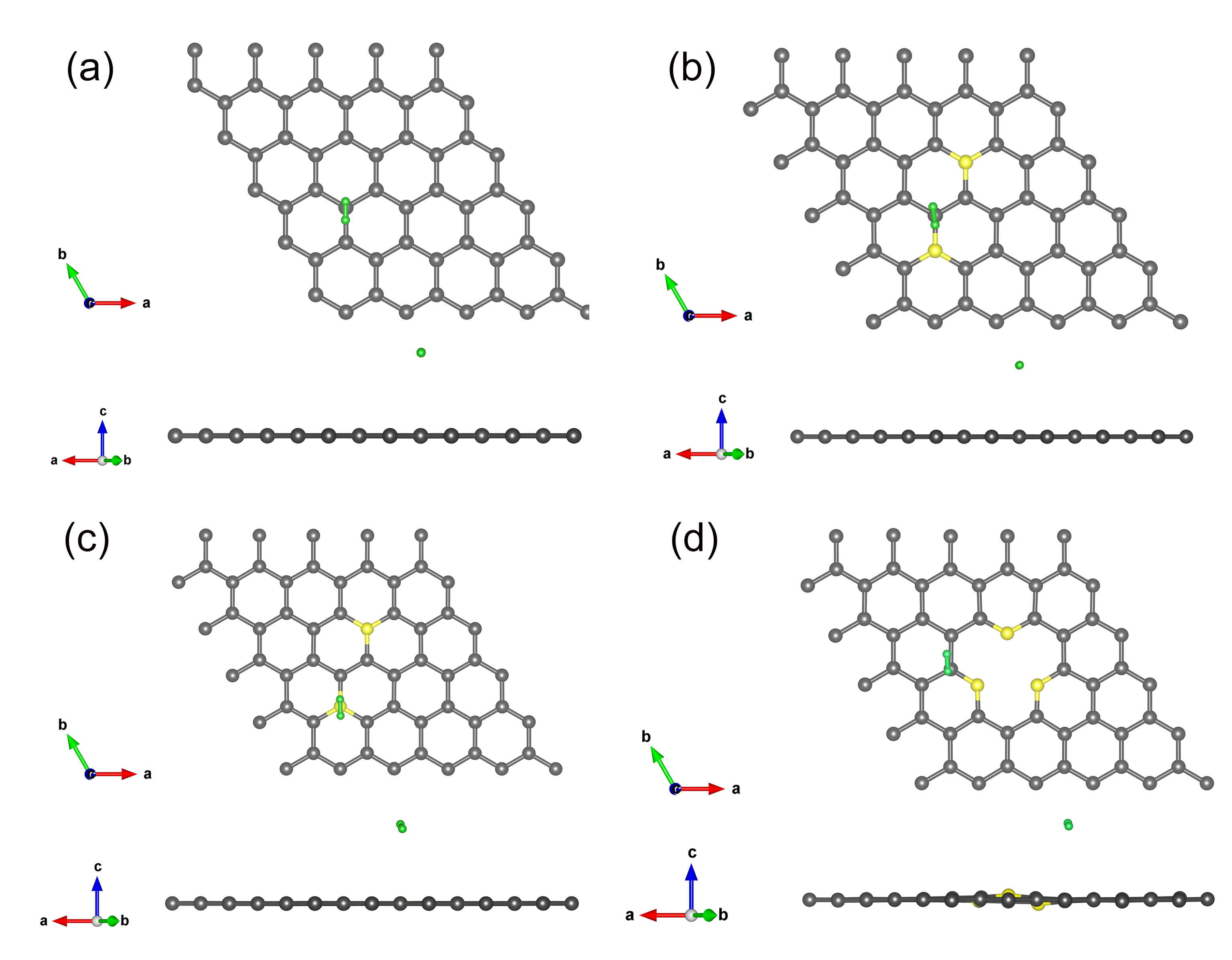


**Fig. S7** Top and side views of optimized geometries of H_2_ adsorbed on **a** graphene, **b** C sites in graphitic N doped graphene, **c** N sites in graphitic N doped graphene, and **d** C sites in pyridinic N doped graphene


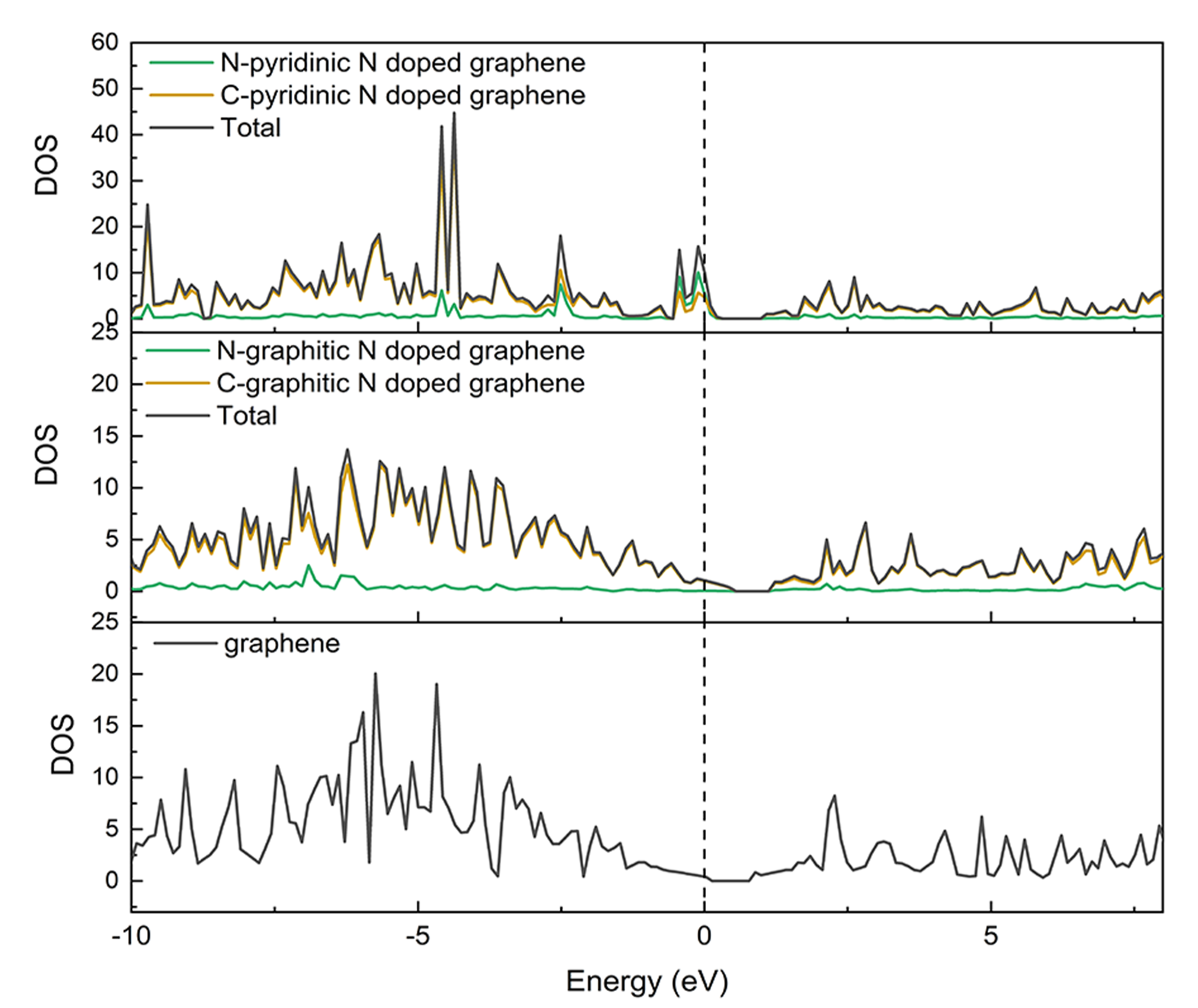


**Fig. S8** Density of States (DOS) for modelled substrates


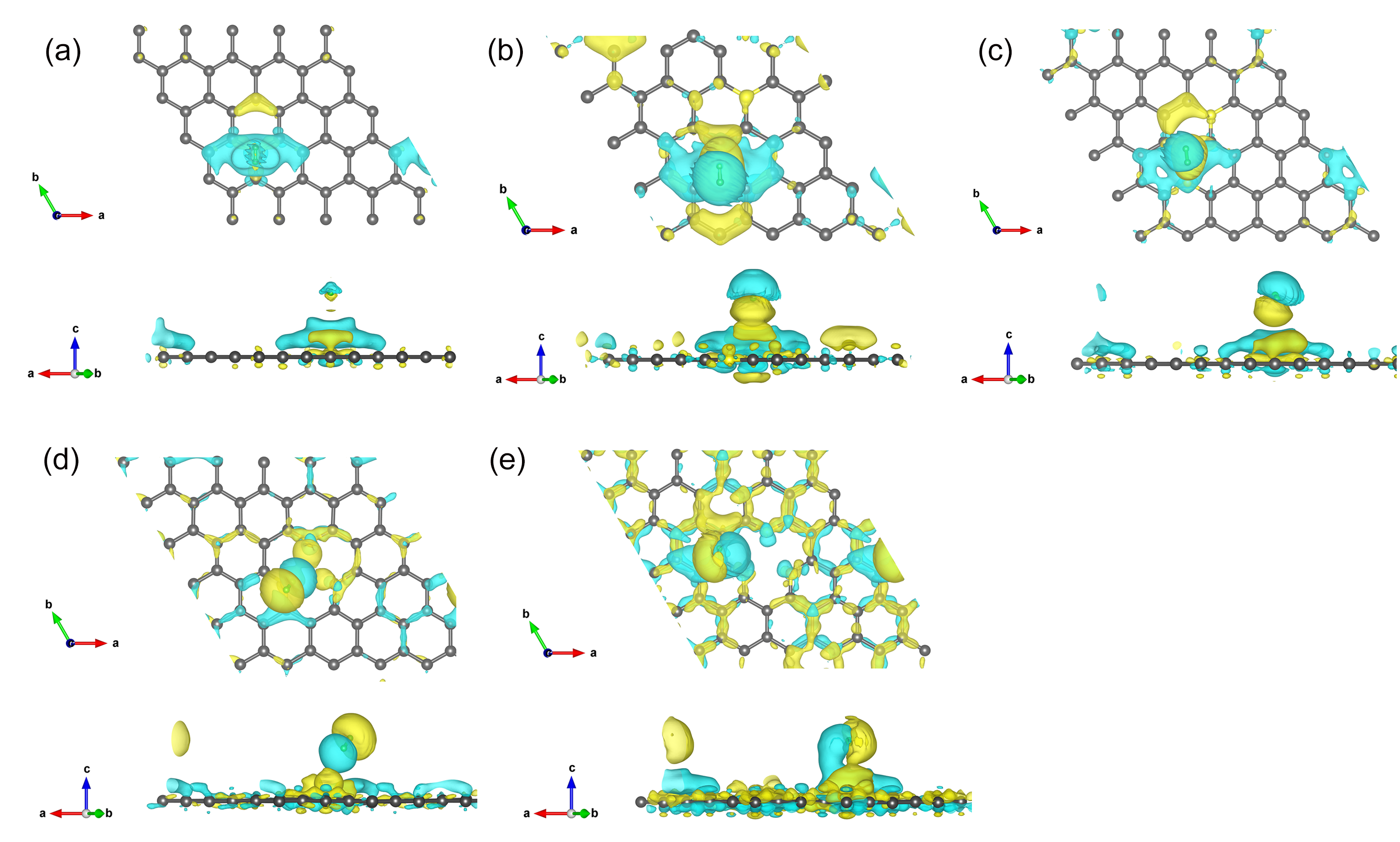


**Fig. S9** Iso-surfaces of charge density difference for H_2_ adsorbed on **a** graphene, **b** N sites in graphitic N doped graphene, **c** C sites in graphitic N doped graphene, **d** N sites in pyridinic N doped graphene, and **e** C sites in pyridinic N doped graphene


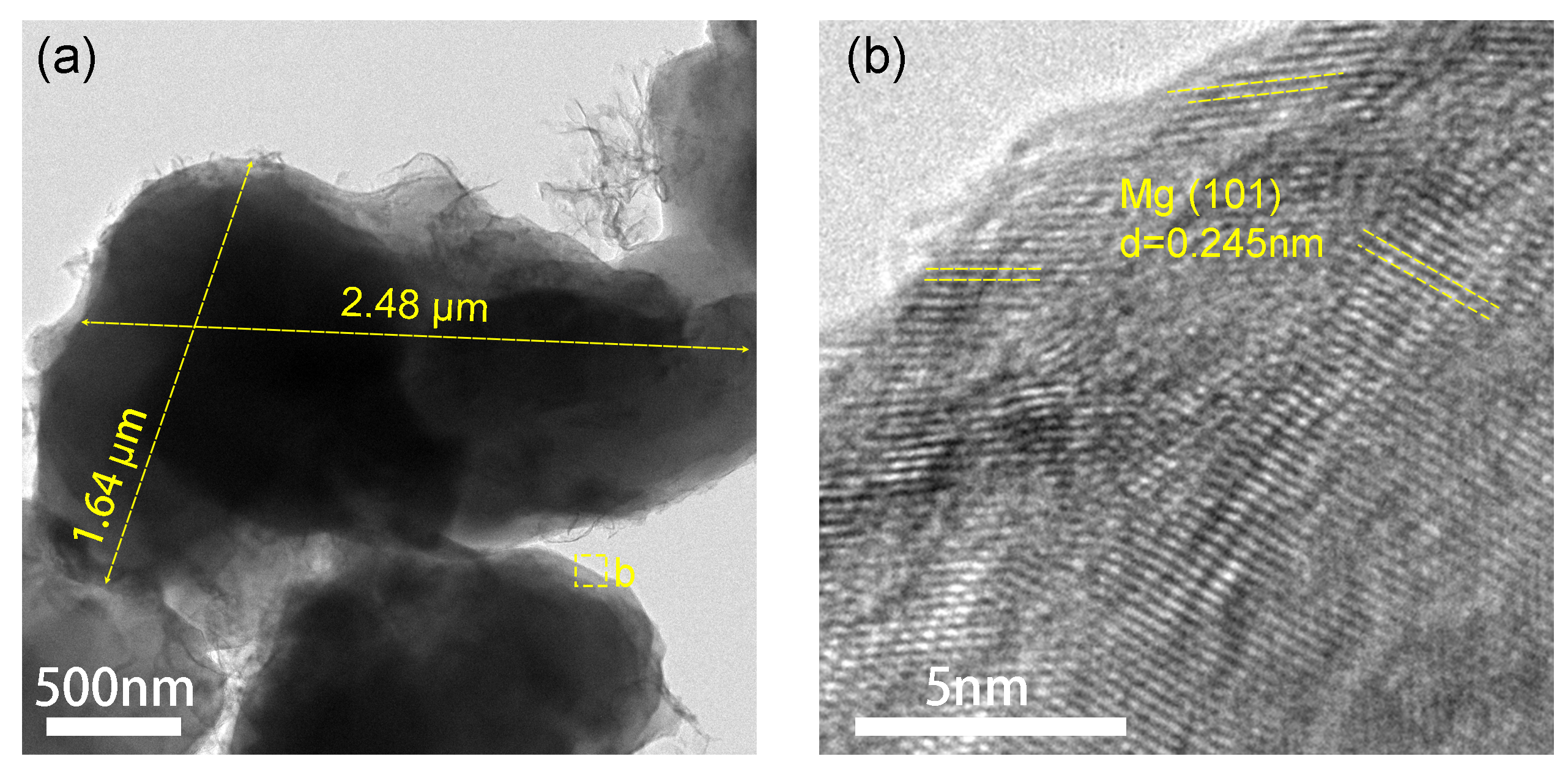


**Fig. S10** **a** Typical TEM and **b** HRTEM images of the as-synthesized pure Mg


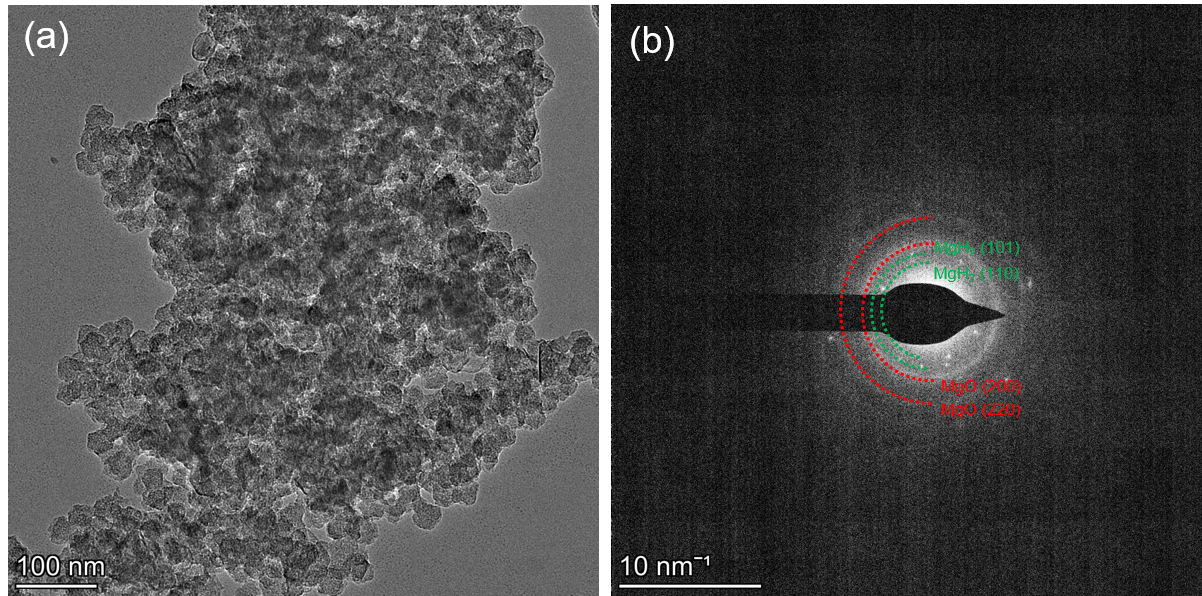


**Fig. S11** **a** TEM image of 60MgH_2_/rN-pC and **b** corresponding SAED pattern


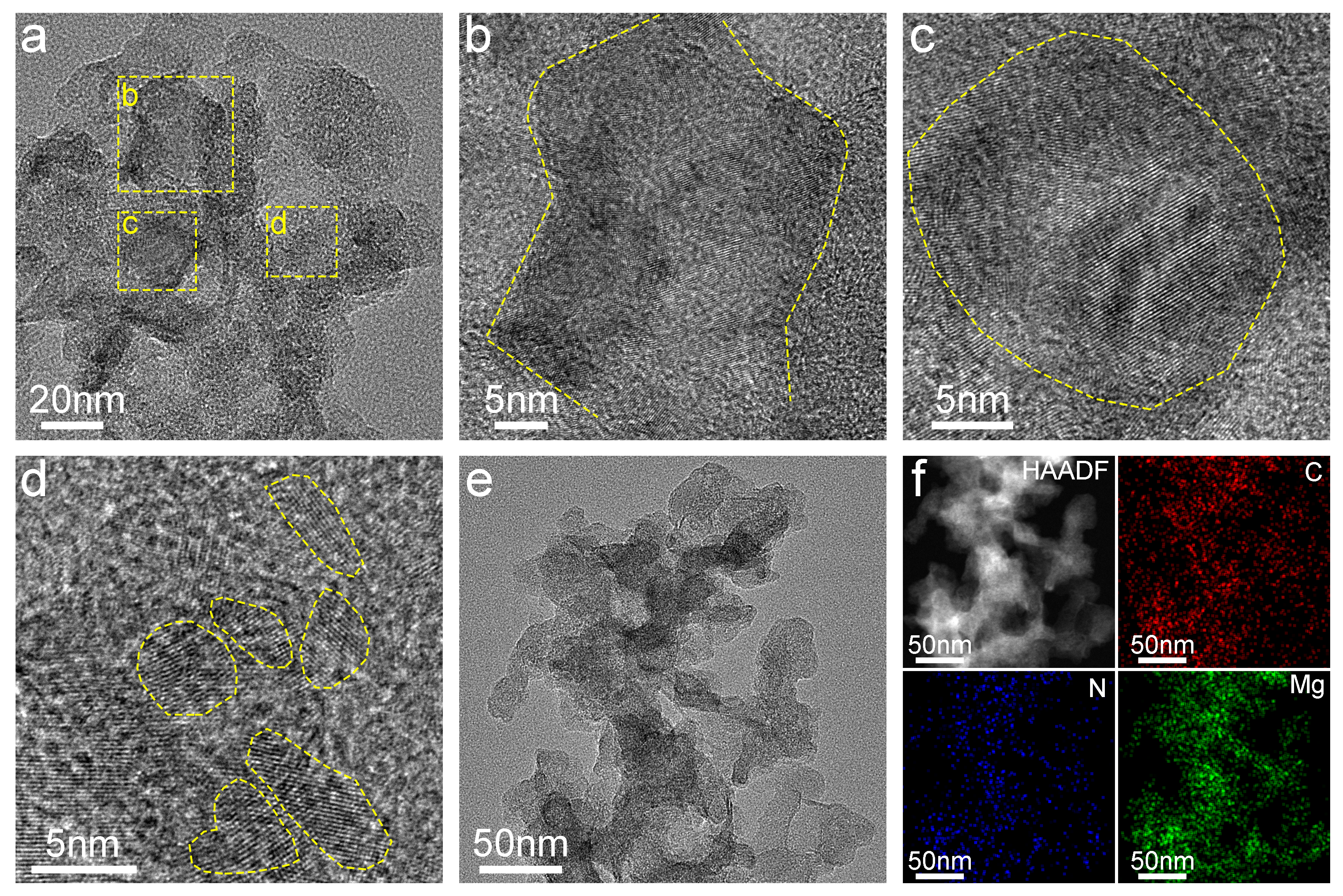


Fig. S12 **a** Typical TEM, and **b, c, d** the corresponding HRTEM images, **e** TEM image and **f** corresponding HAADF image and EDS elemental mapping (C, N, Mg) results of the hydrogenated 60MgH_2_@rN-pC composite after 10 cycles at 275 ^o^C





**Fig. S13** FTIR spectra of rN-pC, pure MgH_2_ and 60MgH_2_/rN-pC


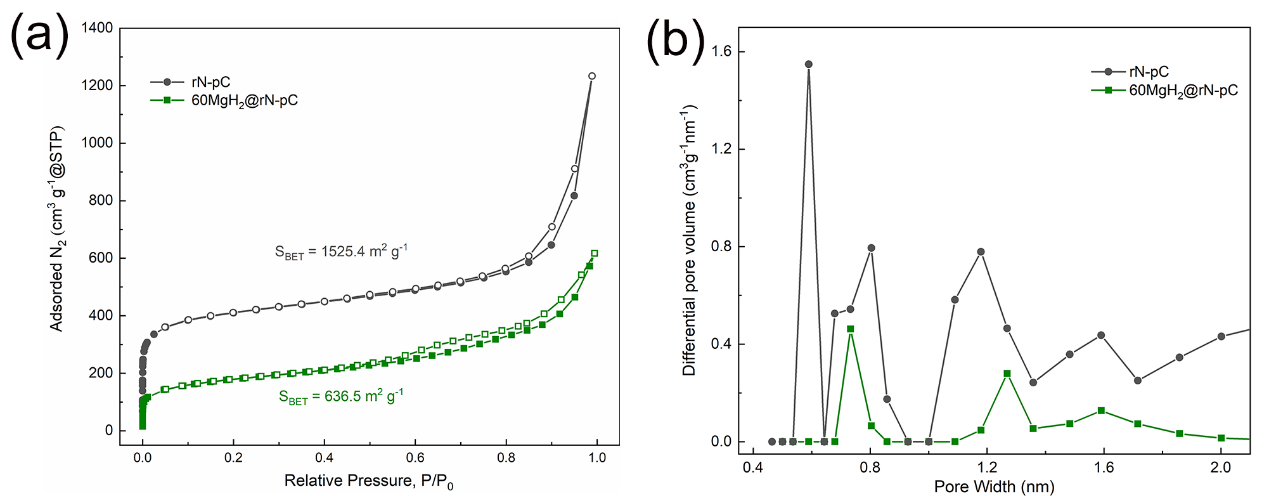


**Fig. S14** **a** N_2_ adsorption/desorption isotherms and **b** pore size distribution of rN-pC and 60MgH_2_@rN-pC


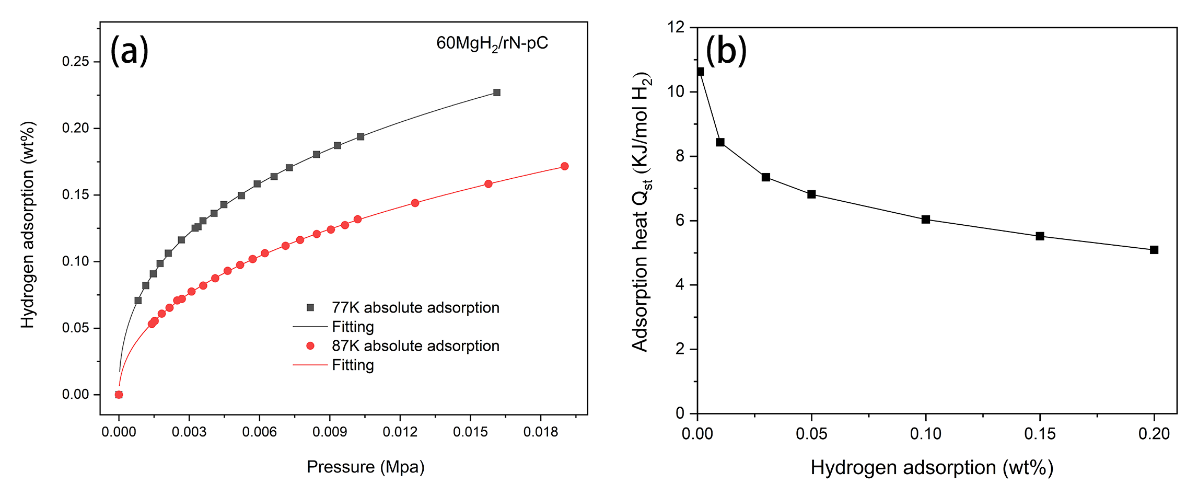


**Fig. S15 a** Experimental H_2_ adsorption of 60MgH_2_/rN-pC at 196 and -186 ^o^C under ultralow pressure and **b** equivalent isosteric heat of H_2_ adsorption fitting of 60MgH_2_/rN-pC


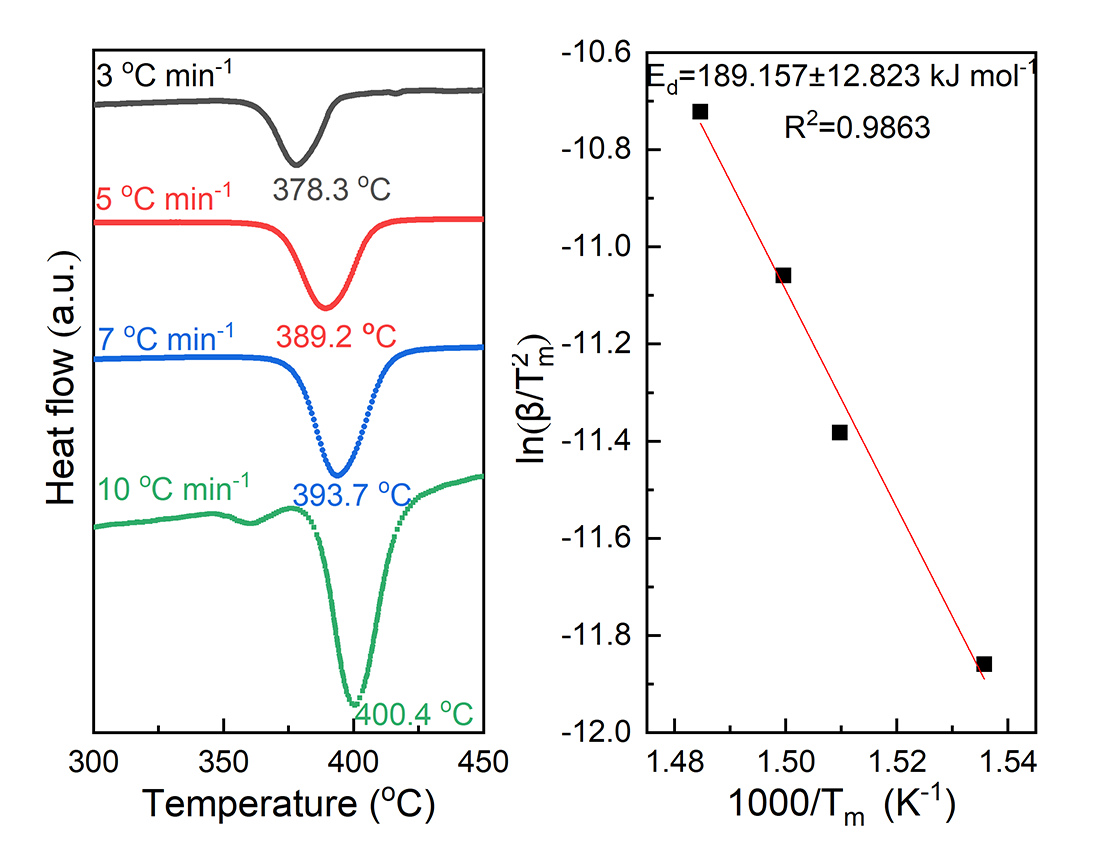


**Fig. S1****6** DSC curves and corresponding Kissinger’s plots of the pure MgH_2_


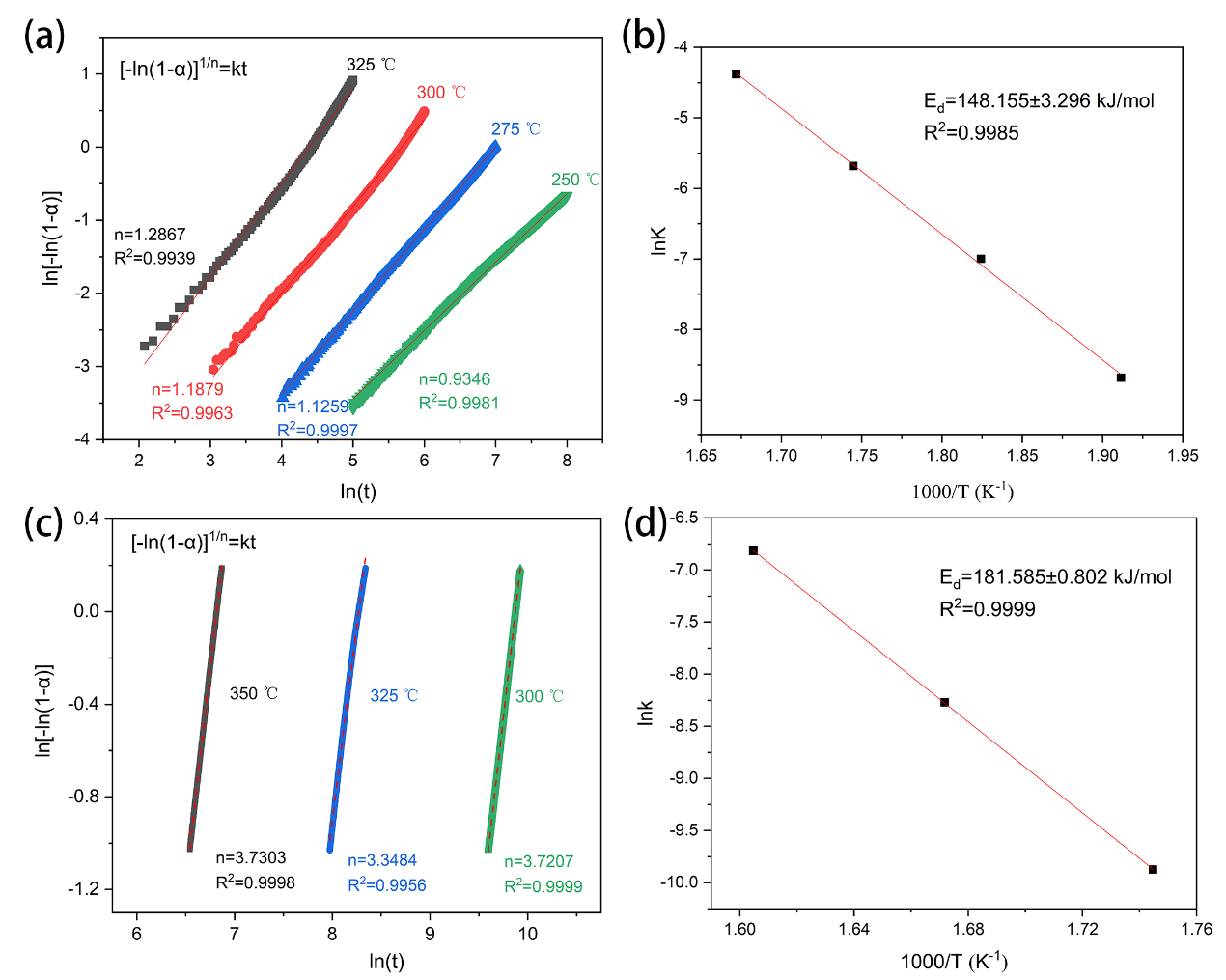


**Fig. S17** JMAK plots and the corresponding lnK-1000/T plots of **a, b** 60MgH_2_/rN-pC and **c, d** pure MgH_2_ for dehydrogenation


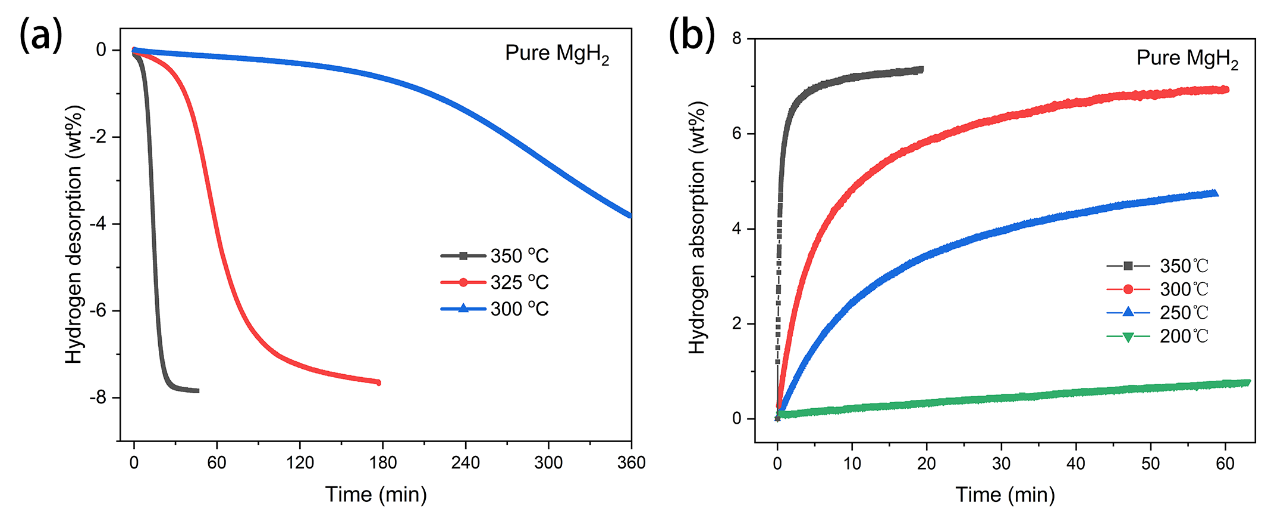


**Fig. S18** Isothermal **a** dehydrogenation and **b** rehydrogenation curves of the pure MgH_2_ at different temperatures


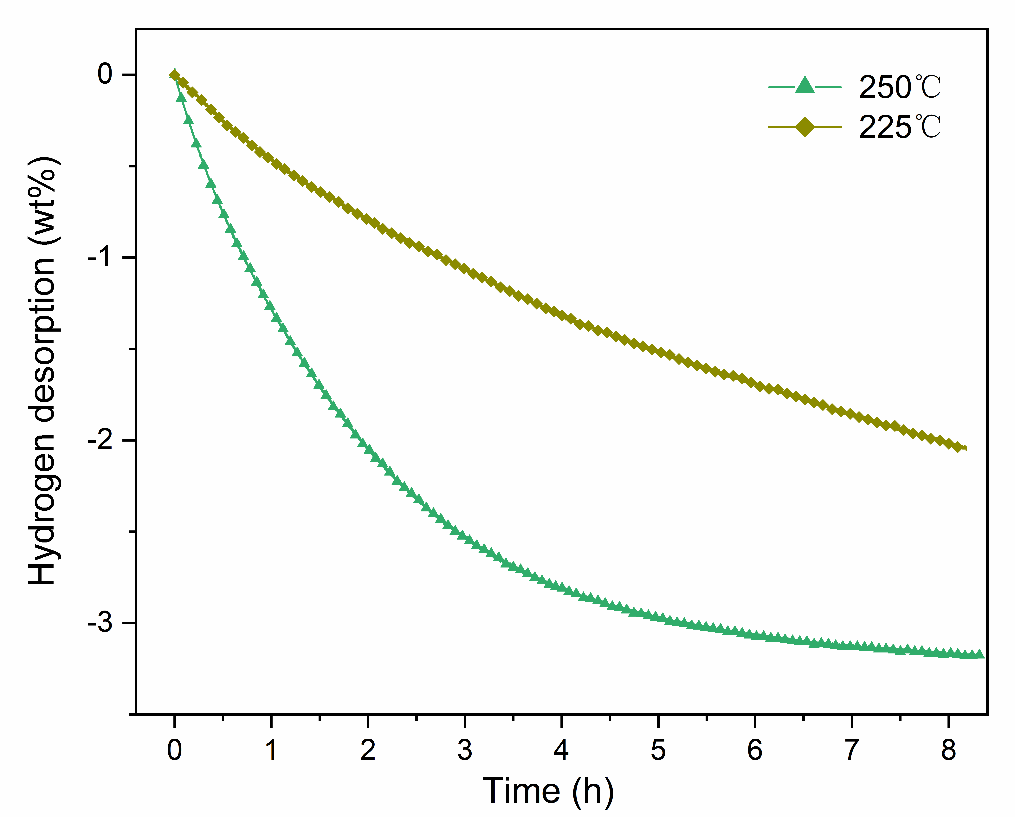


**Fig. S19** Isothermal dehydrogenation curves of 60MgH_2_/rN-pC at 225 ^o^C and 250 ^o^C for a relatively long time (~8h)


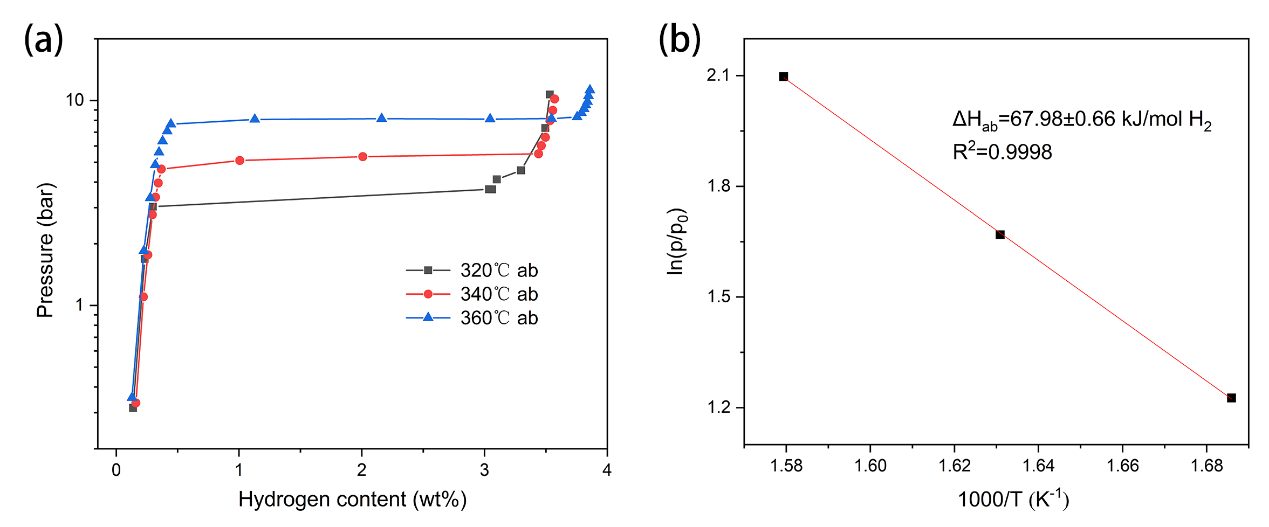


**Fig. S20** **a** PCT curves of 60MgH_2_/rN-pC at different temperatures and corresponding **b** fitting plot


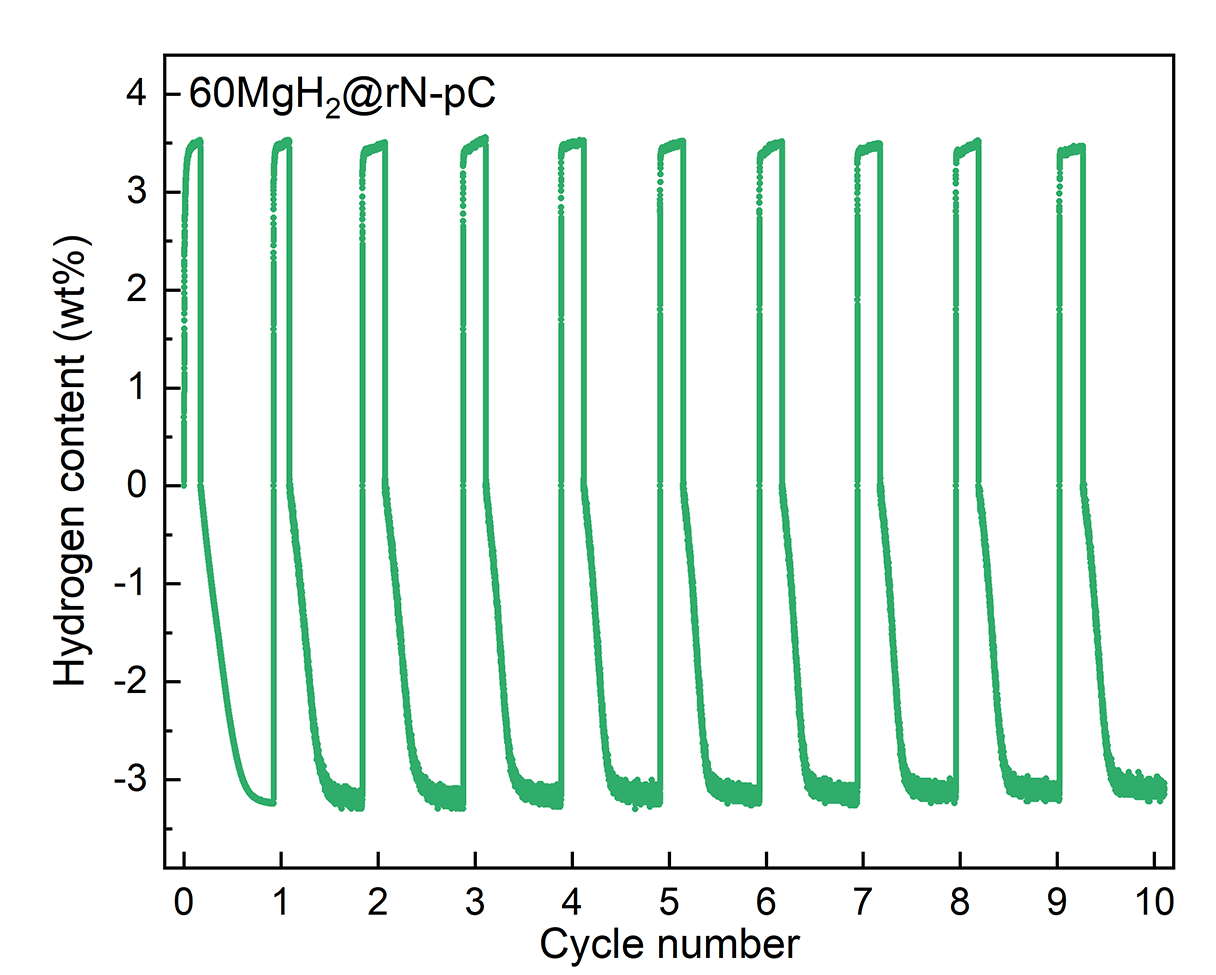


**Fig. S21** Reversible hydrogen absorption/desorption cycling profiles of 60MgH_2_@rN-pC at 275 °C


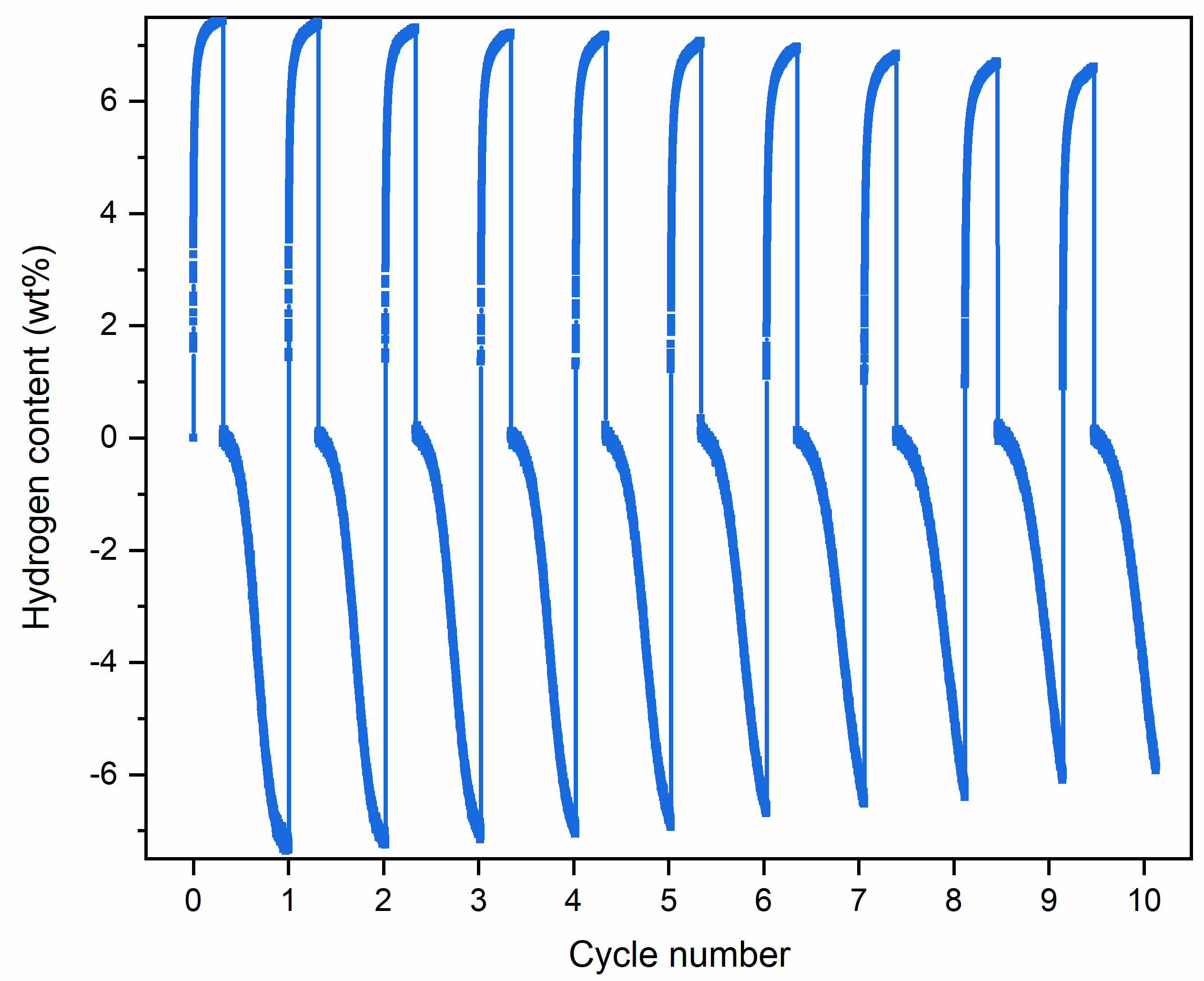


**Fig. S22** Reversible hydrogen absorption/desorption cycling profiles of the pure MgH_2_ at 350 °C





**Fig. S23** Hydrogen desorption curves of 60MgH_2_@rN-pC at 275 ^o^C and the pure MgH_2_ obtained under 350 °C at different cycles


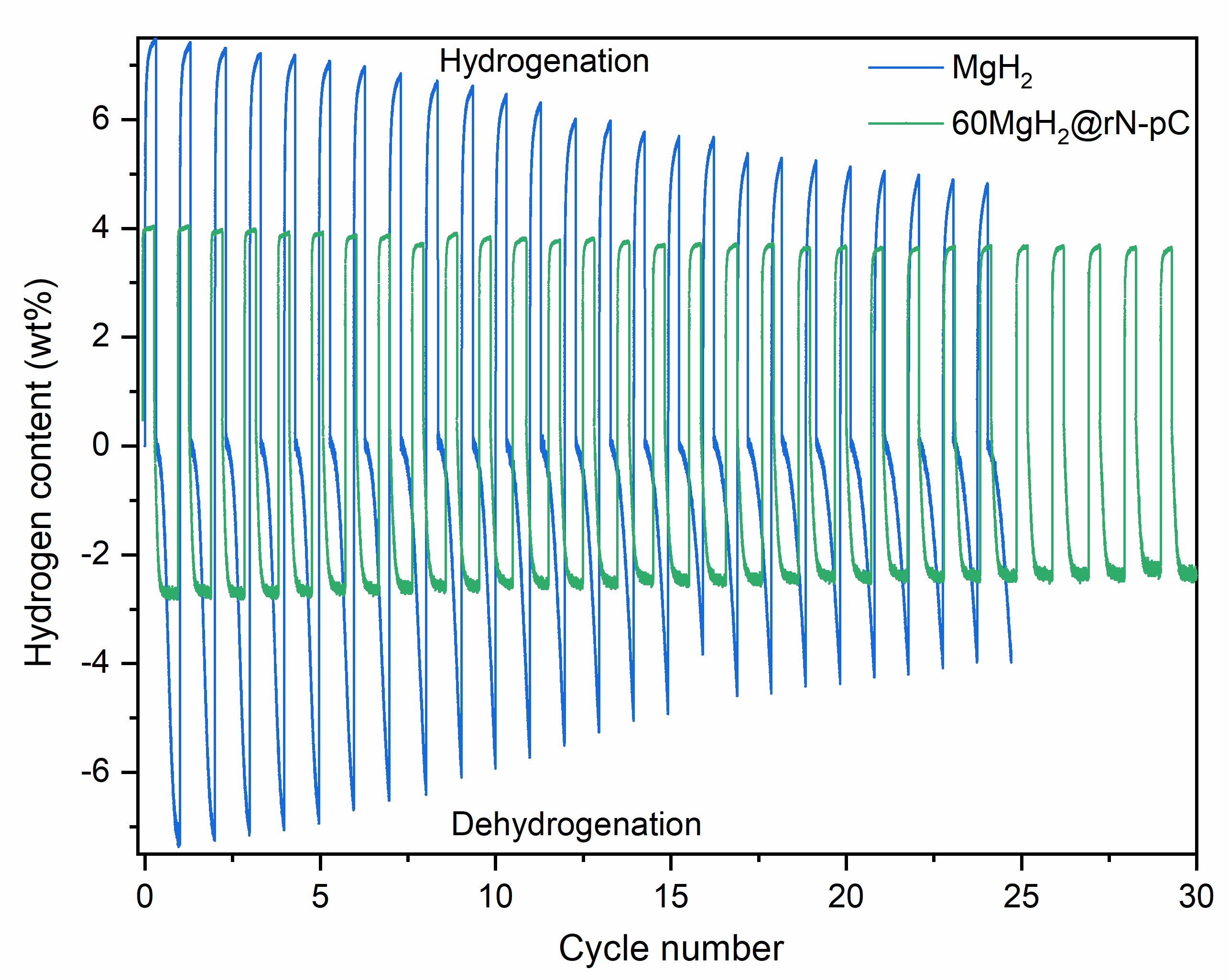


**Fig. S24** Reversible hydrogen absorption/desorption cycling profiles of the pure MgH_2_ at 350 ^o^C for 25 cycles and 60MgH_2_@rN-pC at 300 ^o^C for 30 cycles


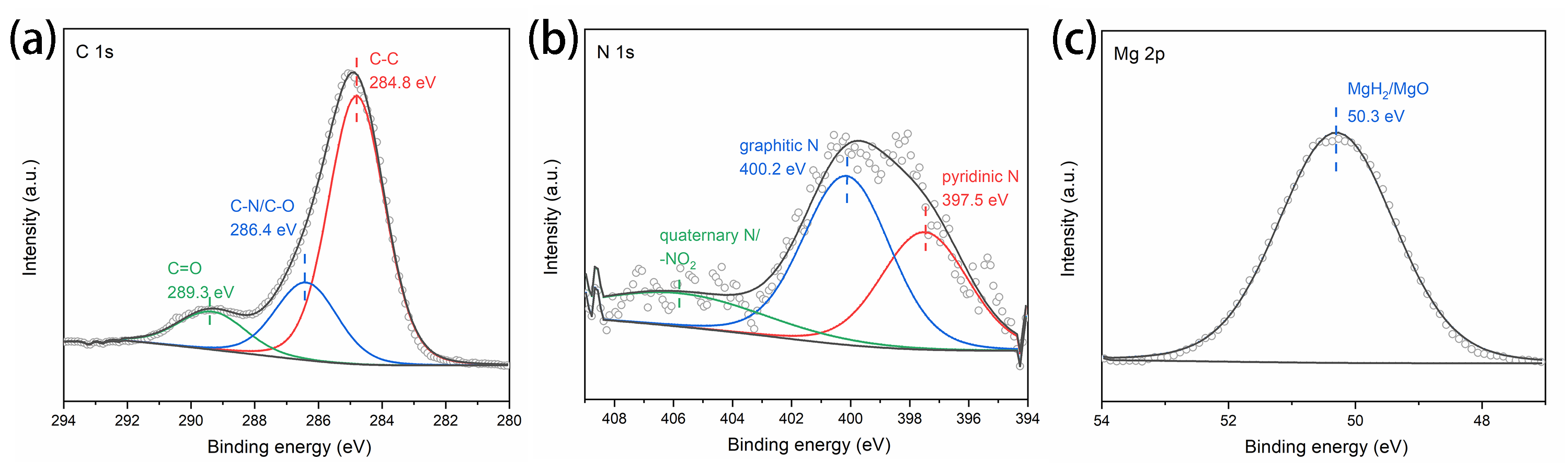


**Fig. S25** High-resolution **a** C 1s, **b** N 1s, and **c** Mg 2p spectra of the hydrogenated 60MgH_2_@rN-pC composite after 10 cycles at 275 ^o^C





**Fig. S26** Continuous desorption curve of the 60MgH_2_@rN-pC composites

About the waste heat to trigger MgH_2_ desorption hypothesis, we provide a quantitative energy balance calculation based on the MgH_2_–solid oxide fuel cells (SOFC) powered prototype car [S1].

Supposing that there is 1 kg of composite material, including 400 g porous carbon and 600 g MgH_2_. According to the references, the specific heat capacities of porous carbon and MgH_2_ can be determined as ~0.8 J g^-1^ K^-1^ [S2, S3] and ~1.4 J g^-1^ K^-1^ [S4], respectively. The working efficiency of SOFC is considered as 60% [S1, S5], and the use of 8 g H_2_ in SOFC would provide waste heat of: 285.8 kJ mol^-1^×8 g/2.0 g mol^-1^×40%=457.3 kJ. If the utilization rate of the waste heat is 60% [S6] and the heat loss rate of the hydrogen storage tank is considered as 20% [S7, S8], it can increase the temperature of the composite from room temperature to: ΔT=457.3 kJ×60%×80%/(0.8 J g^-1^ K^-1^×400 g +1.4 J g^-1^ K^-1^×600 g) =189.2 K. The result indicates that the 60MgH_2_@rN-pC composite can be heated to ~214 ^o^C by the waste heat, which is higher than its onset desorption temperature of 175 ^o^C.


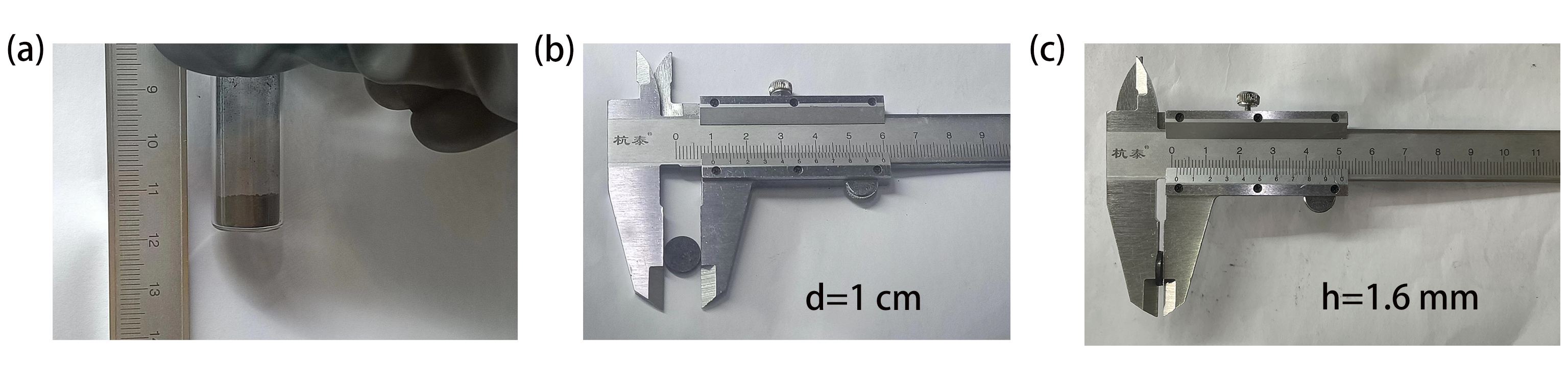


**Fig. S27** Optical photographs of **a** 100mg 60MgH_2_@rN-pC powder and **b, c** the corresponding pellet compacted under 500 MPa from different views


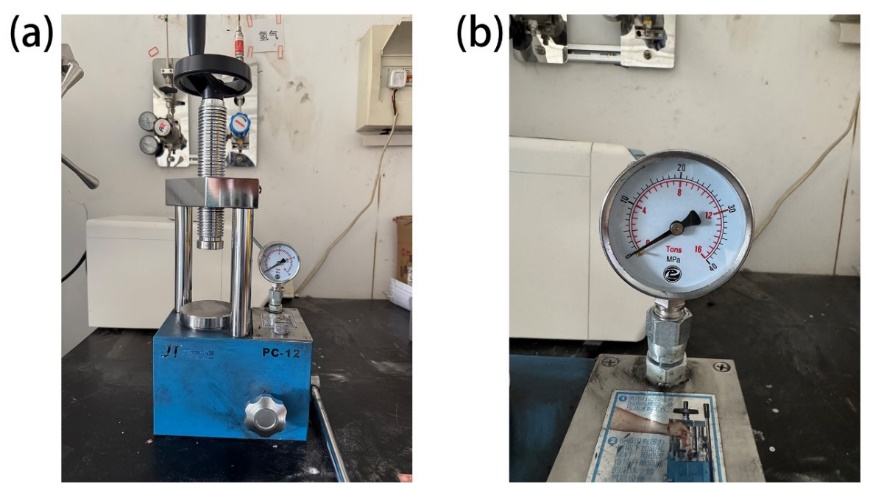


**Fig. S28** The optical photographs of **a** compression machine and its **b** compaction pressure indicator.

The compression machine was a PC-12 type tablet press from Tianjin Jingtuo Company and its effective force range is 0-12 Tons. A cylindrical die with the inner diameter of 10 mm was used for pelletizing. Based on the effective force and the size of die, the effective pressure range is 0-1500 MPa. In this work, a compression pressure of 500 MPa (4 Tons) was employed on the composite pellets.

**Supplementary** **Equations**

**Equation S1** The high-pressure H_2_ isotherms adsorption at 77K and 87K were modelled using a semi-empirical methodology [S9]. The high-pressure H_2_ isotherms were measured by HPSA-auto device, and were fitted using the Tóth equation:

$$m=100\left( \rho_{A}-\frac{PM}{ZRT} \right)\theta v_{p}$$

to represent the filling fraction θ:

$$\theta=\frac{bp}{\left( 1+{bp}^{c} \right)^{1/c}}$$

the compressibility factor Z that accounts for the fact that hydrogen is not an ideal gas at 77 K, Z is approximate as:

$$Z=1-1.378\times{10}^{-3}p+7.02630\times{10}^{-6}p^{2}$$

where ρ_A_ is the density of adsorbate in g cm^-3^, b is the affinity constant in MPa^-1^, c is a heterogeneity parameter, and v_p_ is the pore volume in cm^3^ g^-1^. p is the pressure in MPa, R = 8.314 J mol^-1^ K^-1^ is the molar gas constant, T is the temperature in K and M = 2.01588 g mol^-1^ is the molar mass of H_2_.

**Equation S2** The density functional theory (DFT) is an advanced method to calculate pore size distribution and pore volume based on adsorption isotherm data, especially for the accurate analysis of micropores (<2 nm) and mesopores (2-50 nm) [S10]. The integral equation of isothermal adsorption for the case of distributed pore sizes can be written as the convolution:

$$Q\left( p \right)=\int dHq\left( p,H \right)f(H)$$

Where Q(p) is the total quantity of adsorbate at pressure p, q(p,H), the Kernel function, is the density functional description of the adsorption isotherm for an ideally homo-porous material characterized by pore width H, and f(H) is the desired pore surface area distribution function with respect to H.

**Equation S3** To assess the interaction between H_2_ molecules and the rN-pC, the isosteric heat of adsorption (Q_st_) was calculated using the Clausius-Clapeyron equation [S11], H_2_ isotherm data at T_1_ (77 K) and T_2_ (87 K) were fitted with Langmuir equation.

$$\left( \frac{\delta lnP}{\delta T} \right)_{n}=\frac{Q_{st}}{RT^{2}}$$

$$Q_{st}=-\frac{R\left[ ln\left( P_{T2} \right)-ln\left( P_{T1} \right) \right]}{T_{2}^{-1}-T_{1}^{-1}}$$

**Equation S4** The desorption apparent activation energy (E_a_) was obtained by fitting DSC data using Kissinger method [S12].

$$ln\left( \frac{\beta}{T_{P}^{2}} \right)=-\frac{E_{a}}{RT_{p}}+C$$

where β represents the heating rate, T_p_ refers to the peak temperature, R is the gas constant (R = 8.314 J mol^−1^ K^−1^), and C is a constant.

**Equation S5** The enthalpy of the reaction between hydrogen and Mg was calculated by Van’t Hoff equation [S13].

$$ln\frac{p_{H2}^{eq}}{p^{0}}=\frac{\Delta H^{o}}{RT}-\frac{\Delta S^{o}}{R}$$

Where $p_{H2}^{eq}$ is the equilibrium pressure (plateau pressure) at the temperature T, $p^{0}$ is the reference state (o, 1 bar), $\Delta H^{o}$ and $\Delta S^{o}$ represent the enthalpy and entropy change of the reaction, and R is the gas constant (R = 8.314 J mol^−1^ K^−1^).

**Supplementary** **Tables**

**Table S1** the specific surface area, pore size distribution and pore volume of ZIF-8, N-pC and rN-pC

|  | S_BET_(m^2^ g^-1^) | V_DFT_(mL g^-1^) | S_DFT_(MP) (m^2^ g^-1^) | V_DFT_(MP) (mL g^-1^) |
| --- | --- | --- | --- | --- |
| ZIF-8 | 1877.3 | 0.711 | 1367.9 | 0.666 |
| N-pC | 1146.5 | 0.920 | 921.5 | 0.271 |
| rN-pC | 1525.4 | 1.498 | 1326.3 | 0.447 |

MP: micropore

**Table S2** Semiquantitative atomic fraction of XPS of N-pC and rN-pC (at%)

|  | C | N | O |
| --- | --- | --- | --- |
| N-pC | 95.16 | 2.33 | 2.51 |
| rN-pC | 91.48 | 5.97 | 2.55 |

**Table S3** Semiquantitative atomic fraction of STEM of N-pC and rN-pC (at%)

|  | C | N | O |
| --- | --- | --- | --- |
| N-pC | 94.7 | 1.36 | 3.98 |
| rN-pC | 94.4 | 2.06 | 3.52 |

**Table S4** The high-pressure H_2_ isotherms adsorption at 77K and 87K were modelled using a semi-empirical methodology for rN-pC and 60MgH_2_/rN-pC

| Sample | ρ_A_  (g cm^-3^) | b  (MPa^-1^) | c | v_p_  (cm^3^ g^-1^) | R^2^ | Max excess adsorption (wt%) |
| --- | --- | --- | --- | --- | --- | --- |
| rN-pC | 0.02629 | 532.995 | 0.29197 | 1.77791 | 0.9983 | 2.49 |
| 60MgH_2_/rN-pC | 0.04542 | 3407.31 | 0.20782 | 0.34015 | 0.9995 | 0.66 |

**Supplementary References**

1. X. Wang, L. Shao, S. Hu, Z. Li, H. Guo et al., A techno-economic study of photovoltaic-solid oxide electrolysis cell coupled magnesium hydride-based hydrogen storage and transportation toward large-scale applications of green hydrogen. Energy Environ. Sci. **17**(22), 8429–8456 (2024). <https://doi.org/10.1039/D4EE04224G>
2. M. Amirul Islam, K. Thu, B. B. Saha, Specific heat capacity of mangrove and waste palm trunk in raw, carbonized and activated form, IEICES, **4**,151-152 (2018).
3. M. Kano, M. Momota, T. Okabe, K. Saito, Specific heat capacity of new porous carbon materials: Woodceramics. Thermochim. Acta **292**(1–2), 175–177 (1997). <https://doi.org/10.1016/S0040-6031(96)03110-3>
4. P. Larpruenrudee, N.S. Bennett, Y. Gu, R. Fitch, M.S. Islam, Design optimization of a magnesium-based metal hydride hydrogen energy storage system. Sci. Rep. **12**(1), 13436 (2022). <https://doi.org/10.1038/s41598-022-17120-3>
5. J. Rechberger, A. Kaupert, J. Hagerskans, L. Blum, Demonstration of the first European SOFC APU on a heavy duty truck. Transp. Res. Procedia **14**, 3676–3685 (2016). <https://doi.org/10.1016/j.trpro.2016.05.442>
6. Metropolitan.fi. Data Center Heats Homes: Yandex Sells Server Waste Energy to District Heating.
7. X. Lin, C.-L. Yin, L. Ren, Y. Li, Z. Li et al., A one- and three-dimensional coupled model and simulation investigation for the large-scale oil-heating type Mg-based hydrogen storage tank. Chem. Eng. J. **472**, 144943 (2023). <https://doi.org/10.1016/j.cej.2023.144943>
8. L. Shao, X. Lin, L. Bian, Y. Wang, S. Hu et al., Engineering control strategy of hydrogen gas direct-heating type Mg-based solid state hydrogen storage tanks: a simulation investigation. Appl. Energy **375**, 124134 (2024). <https://doi.org/10.1016/j.apenergy.2024.124134>
9. V.P. Ting, A.J. Ramirez-Cuesta, N. Bimbo, J.E. Sharpe, A. Noguera-Diaz et al., Direct evidence for solid-like hydrogen in a nanoporous carbon hydrogen storage material at supercritical temperatures. ACS Nano **9**(8), 8249–8254 (2015). <https://doi.org/10.1021/acsnano.5b02623>
10. M.L. Occelli, J.P. Olivier, J.A. Perdigon-Melon, A. Auroux, Surface area, pore volume distribution, and acidity in mesoporous expanded clay catalysts from hybrid density functional theory (DFT) and adsorption microcalorimetry methods. Langmuir **18**(25), 9816–9823 (2002). <https://doi.org/10.1021/la020567o>
11. S. Poyet, S. Charles, Temperature dependence of the sorption isotherms of cement-based materials: Heat of sorption and Clausius–Clapeyron formula. Cem. Concr. Res. **39**(11), 1060–1067 (2009). <https://doi.org/10.1016/j.cemconres.2009.07.018>
12. R.L. Blaine, H.E. Kissinger, Homer Kissinger and the Kissinger equation. Thermochim. Acta **540**, 1–6 (2012). <https://doi.org/10.1016/j.tca.2012.04.008>
13. J.F. Stampfer Jr, C.E. Holley Jr, J.F. Suttle, The magnesium-hydrogen System^1-3^. J. Am. Chem. Soc. **82**(14), 3504–3508 (1960). <https://doi.org/10.1021/ja01499a006>
